# The gut microbiome modifies the associations of short- and long-term physical activity with body weight changes

**DOI:** 10.1186/s40168-023-01542-w

**Published:** 2023-05-30

**Authors:** Kai Wang, Raaj S. Mehta, Wenjie Ma, Long H. Nguyen, Dong D. Wang, Andrew R. Ghazi, Yan Yan, Laila Al-Shaar, Yiqing Wang, Dong Hang, Benjamin C. Fu, Shuji Ogino, Eric B. Rimm, Frank B. Hu, Rachel N. Carmody, Wendy S. Garrett, Qi Sun, Andrew T. Chan, Curtis Huttenhower, Mingyang Song

**Affiliations:** 1 Department of Epidemiology, Harvard T.H. Chan School of Public Health, 667 Huntington Avenue, Kresge 906A, Boston, MA 02115 USA; 2 Clinical and Translational Epidemiology Unit, Massachusetts General Hospital and Harvard Medical School, Boston, MA USA; 3 Division of Gastroenterology, Massachusetts General Hospital and Harvard Medical School, Boston, MA USA; 4 Broad Institute of MIT and Harvard, Cambridge, MA USA; 5 Department of Biostatistics, Harvard T.H. Chan School of Public Health, Boston, MA USA; 6 Harvard Chan Microbiome in Public Health Center, Harvard T.H. Chan School of Public Health, Boston, MA USA; 7 Department of Nutrition, Harvard T.H. Chan School of Public Health, Boston, MA USA; 8 Department of Medicine, Channing Division of Network Medicine, Brigham and Women’s Hospital and Harvard Medical School, Boston, MA USA; 9 Department of Epidemiology and Biostatistics, International Joint Research Center On Environment and Human Health, Center for Global Health, School of Public Health, Nanjing Medical University, Nanjing, China; 10 Jiangsu Key Lab of Cancer Biomarkers, Prevention and Treatment, Collaborative Innovation Center for Cancer Medicine, Nanjing Medical University, Nanjing, China; 11 Department of Oncologic Pathology, Dana-Farber Cancer Institute and Harvard Medical School, Boston, MA USA; 12 Department of Pathology, Program in MPE Molecular Pathological Epidemiology, Brigham and Women’s Hospital and Harvard Medical School, Boston, MA USA; 13 Department of Human Evolutionary Biology, Harvard University, Cambridge, MA USA; 14 Department of Immunology and Infectious Diseases, Harvard T.H. Chan School of Public Health, Boston, MA USA; 15 Department of Medical Oncology, Dana-Farber Cancer Institute and Harvard Medical School, Boston, MA USA

## Abstract

**Background:**

The gut microbiome regulates host energy balance and adiposity-related metabolic consequences, but it remains unknown how the gut microbiome modulates body weight response to physical activity (PA).

**Methods:**

Nested in the Health Professionals Follow-up Study, a subcohort of 307 healthy men (mean[SD] age, 70[4] years) provided stool and blood samples in 2012–2013. Data from cohort long-term follow-ups and from the accelerometer, doubly labeled water, and plasma biomarker measurements during the time of stool collection were used to assess long-term and short-term associations of PA with adiposity. The gut microbiome was profiled by shotgun metagenomics and metatranscriptomics. A subcohort of 209 healthy women from the Nurses’ Health Study II was used for validation.

**Results:**

The microbial species *Alistipes putredinis* was found to modify the association between PA and body weight. Specifically, in individuals with higher abundance of *A. putredinis*, each 15-MET-hour/week increment in long-term PA was associated with 2.26 kg (95% CI, 1.53–2.98 kg) less weight gain from age 21 to the time of stool collection, whereas those with lower abundance of *A. putredinis* only had 1.01 kg (95% CI, 0.41–1.61 kg) less weight gain (*p*_interaction_ = 0.019). Consistent modification associated with *A. putredinis* was observed for short-term PA in relation to BMI, fat mass%, plasma HbA1c, and 6-month weight change. This modification effect might be partly attributable to four metabolic pathways encoded by *A. putredinis*, including folate transformation, fatty acid β-oxidation, gluconeogenesis, and stearate biosynthesis.

**Conclusions:**

A greater abundance of *A. putredinis* may strengthen the beneficial association of PA with body weight change, suggesting the potential of gut microbial intervention to improve the efficacy of PA in body weight management.

Video Abstract

**Supplementary Information:**

The online version contains supplementary material available at 10.1186/s40168-023-01542-w.

## Introduction

Overweight and obesity are global health problems [1, 2] and have been associated with increased risk of morbidity and mortality and decreased odds of healthy aging [3]. The gut microbiome has been increasingly recognized to play important roles in modulating host energy balance and adiposity-related metabolic consequences [4, 5]. Physical activity (PA) increases energy expenditure and thus is often prescribed for body weight management [6]. Although weight loss is often reported after PA intervention [7, 8], PA does not always result in expected weight loss and there is high variability in body weight responses to PA [9–17].

In the growing field of precision medicine, there is great interest in identifying whether specific features of the gut microbiome may modulate the effect of PA on body weight. Certain gut microbial features, e.g., *Blautia wexlerae* [18], *Bacteroides dorei* [18], *Bacteroides cellulosilyticus* [19], Prevotella-to-Bacteroides ratio [20], and bacterial metabolites, e.g., choline and L-carnitine [21], have been recently reported to associate body weight response to dietary interventions. A recent study identified gut microbiota as an important determinant of the response to 12-week’s PA intervention in the improvement of glucose metabolism and insulin sensitivity among overweight individuals with prediabetes [22]. However, few studies have examined how the gut microbiome may modulate body weight response to PA in the generally healthy population.

Therefore, in the current study, we leveraged the integrated epidemiologic and shotgun metagenomic and metatranscriptomic data in two cohorts and examined the effect modification of the gut microbiome on the relationships of recent and long-term (26-year) PA with multiple body weight measures, including body mass index (BMI), fat mass percentage, short (6-month)- and long-term (early-to-middle adulthood) body weight change, as well as plasma biomarkers of high-sensitivity C-reactive protein (CRP) and hemoglobin A1c (HbA1c). We conducted the primary analysis among a subcohort of 307 men in the long-running Health Professionals Follow-up Study (HPFS) and validated the main findings among 209 women from the Nurses’ Health Study (NHS) II.

## Results

### Overview of the discovery cohort

**Fig. 1:**
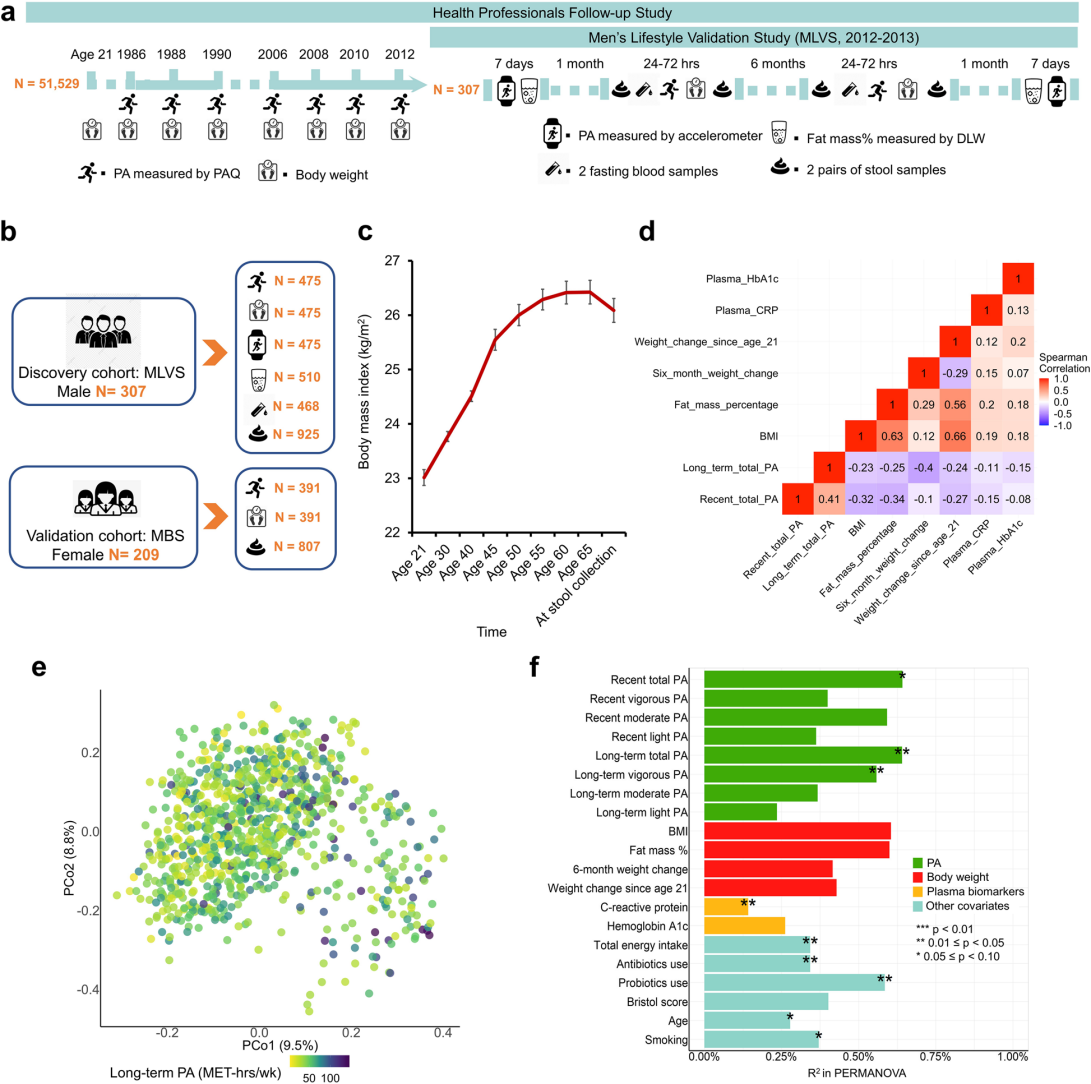
Study design for linking physical activity (PA), body weight measures, plasma biomarkers, and the gut microbiome in the Men’s Lifestyle Validation Study (MLVS) and associations with overall gut microbiome configuration.** a** To associate the gut microbiome with PA and body weight measures, we profiled stool metagenomes, metatranscriptomes, and blood biomarkers from the MLVS. The MLVS is a sub-study of the Health Professionals Follow-up Study (HPFS), an ongoing prospective cohort of 51,529 men. The HPFS has repeatedly collected PA and body weight information using validated questionnaires and health-related information since 1986. In the period 2012–2013, the MLVS collected stool samples at up to four time points per individual, blood samples at up to two time points, and additional PA information using accelerometer and body weight information using doubly labeled water from 307 participants. We applied MetaPhlAn 2 and HUMAnN 2 to perform taxonomic and functional profiling from stool shotgun metagenomes and metatranscriptomes. Plasma biomarkers of inflammation (high-sensitivity C-reactive protein, CRP) and glucose homeostasis (hemoglobin A1c, HbA1c) were measured using standard methods. We employed generalized linear mixed-effects regression models to account for within-subject correlation due to repeated sampling and occasional missing data. **b** Discovery cohort, validation cohort, and main exposure and outcome variables used in this study. **c** Mean BMI at different ages and the time of stool collection. The error bars represent standard deviations. **d** Spearman correlation between main exposure and outcome variables. **e** Principal coordinate analysis of all samples using species-level Bray–Curtis dissimilarity. **f** Proportion of variation in taxonomy explained by PA measures, body weight measures, plasma biomarkers, and covariables as quantified by two-sided permutational multivariate analysis of variance (based on species-level Bray–Curtis dissimilarity)

In the Men’s Lifestyle Validation Study (MLVS), a sub-study of the HPFS, a total of 925 metagenomes, 340 metatranscriptomes, and 468 plasma biomarker measurements were included in the analyses (Fig. 1b). Mean (standard deviation, SD) age at stool collection was 70 (4) years. At the time of stool collection, participants with higher recent PA levels were younger, more likely to have lower BMI, lower fat mass percentage, less weight gain from age 21 to age at stool collection (mean difference [SD] = 50 [4] years), and lower plasma CRP and HbA1c levels (Supplementary Table 1). From age 21 to the time at stool collection, participants gained a mean weight (SD) of 8 (10) kg; the mean BMI (SD) increased from 23.0 (0.2) kg/m^2^ and peaked at age 65 with 26.4 (0.2) kg/m^2^ (Fig. 1c). Recent and long-term total PA and PA by intensity were correlated at weak to moderate magnitude (*r*, ranging from − 0.44 to 0.68, Fig. 1d and Supplementary Table 2). PA was weakly correlated with energy-adjusted macronutrient and total calorie intakes (|*r*|< 0.25, Supplementary Fig. 1).

### A modest but distinguishable association between PA and the gut microbiome

A total of 139 microbial species were included in the analysis after quality control (“Statistical analysis” section). Neither long-term PA (Fig. 1e) nor recent PA level (Supplementary Fig. 2) was a major driver of the variation in overall gut microbial community structure. We did not observe a significant association of PA level with microbial diversity (Shannon index, *p*_trend_ = 0.79, Supplementary Fig. 3). Omnibus testing with permutational multivariate analysis of variance (PERMANOVA) revealed that long-term PA contributed 0.64% (R^2^, *p* = 0.043) of variation in the taxonomic structure (Fig. 1f).

**Fig. 2:**
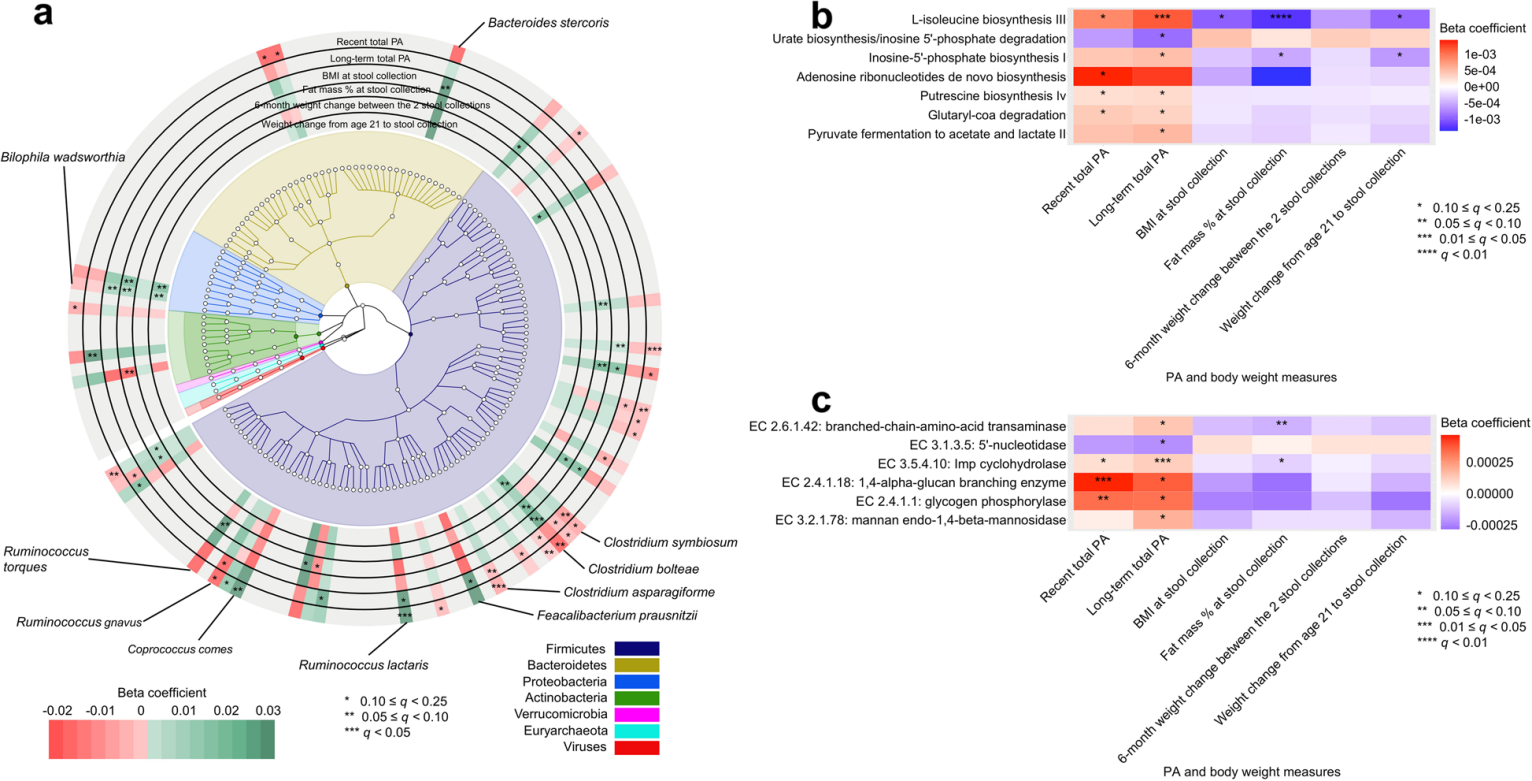
Associations of physical activity (PA) and body weight measures with individual gut microbial species and pathway abundances in the Men’s Lifestyle Validation Study (MLVS).** a** Significant associations of recent and long-term total PA and body weight measure with microbial species (*q* ≤ 0.25). The *q* values (false discovery rate adjusted *p* value) were calculated using the Benjamini–Hochberg method with a target rate of 0.25. This plot shows associations of the factors with specific microbial species overlaid onto their taxonomy. The red-to-green gradient in the outer rings represents the magnitude and direction of the associations between the factors and species’ abundances. The colors of the innermost ring and phylogenetic trees differentiate major phyla. All models included each participant’s identifier as random effects and simultaneously adjusted for age, smoking, Alternative Healthy Eating Index (AHEI), total energy intake, probiotic use, antibiotic use, and Bristol stool scale. **b, c** Associations of recent and long-term total PA and body weight measures with microbial functions (as MetaCyc pathways and EC enzymes). Beta coefficients were derived from multivariable-adjusted generalized linear mixed-effects regression models as above, with multiple comparison adjustment also as above. All the analyses in these panels were conducted based on all 925 metagenomes collected from 307 participants. All the statistical tests were two-sided

We found 45 species-level features from four phyla to be associated with at least one of the measures of PA, body weight, and biomarkers (*q* < 0.25; Supplementary Fig. 4). Generally, associations of the species with PA were in opposite direction to their associations with body weight, e.g., species in the *Clostridium* genus and short-chain fatty acid (SCFA)-producing bacteria, such as *Faecalibacterium prausnitzii* and *Coprococcus comes* (Fig. 2a). Among PA by intensity, vigorous PA was the major driving force of the associations of total PA with the microbial features (Supplementary Fig. 4).

A total of 35 MetaCyc pathways (Supplementary Fig. 5) and 99 Enzyme Commission (ECs) (Supplementary Fig. 6) were associated with at least one of the measures of PA, body weight, and biomarkers. Concordant with the fact that PA increases energy expenditure, we found enrichments of microbial functions for glucose metabolism in individuals with higher PA level. For example, PA, particularly long-term total and vigorous PA, was positively associated with the abundance of L-isoleucine biosynthesis III (PWY-5103, Fig. 2b) and an enzyme in the pathway (EC 2.6.1.42: branched-chain-amino-acid transaminase, Fig. 2c). PA was also found to be positively associated with SCFA-producing microbial pathways, such as pyruvate fermentation to acetate and lactate II (PWY-5100).

These results suggest that PA has a limited effect on overall gut microbial community structure but may be important for some specific taxonomic features and microbial pathways mainly involved in glucose metabolism and SCFA production.

### Differential body weight response to PA by the gut microbiome

We analyzed long-term PA as the main exposure and weight change from age 21 to stool collection as the main outcome, and secondarily examined recent PA in relation to current BMI, fat mass percentage, 6-month weight change, plasma CRP, and HbA1c. Except for the association with CRP, we found all the other associations were significantly modified by the 1st or 2nd principal coordinate loading scores (PCo1 or PCo2) of the overall gut community structure (*p*_interaction_ ≤ 0.035) (Supplementary Fig. 7).

**Fig. 3:**
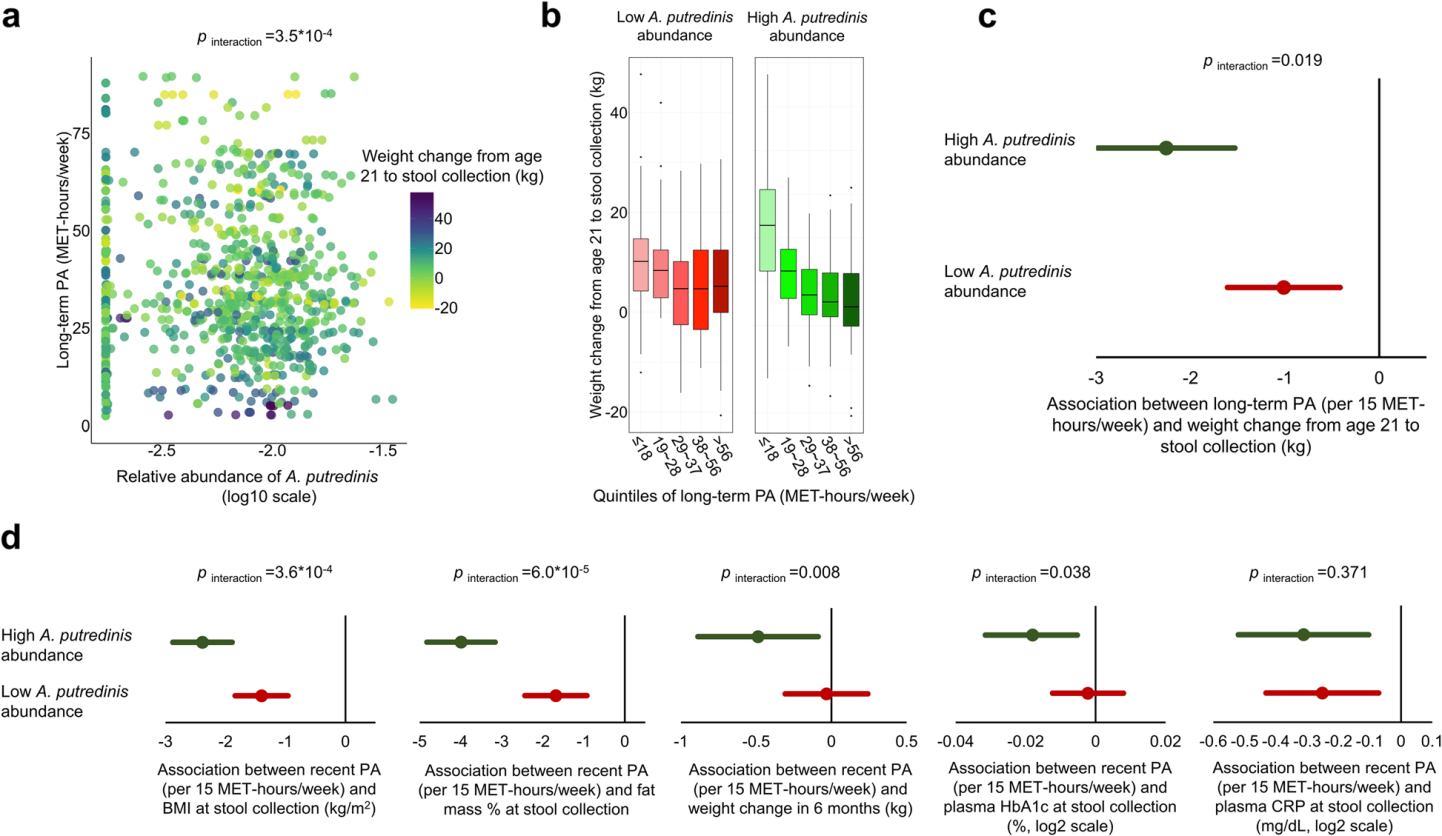
*Alistipes putredinis* abundance modifies the associations of physical activity (PA) measures with body weight measures and plasma biomarkers. Median abundance of *A. putredinis* was used as cutoff for low and high level. A high abundance of *A. putredinis* significantly strengthened the associations of PA with weight loss/less weight gain from age 21 to stool collection (**a**–**c**), lower body mass index (BMI) at stool collection, lower fat mass percentage at stool collection, weight loss/less weight gain in 6 months, and lower plasma hemoglobin A1c (HbA1c) at stool collection, but not with plasma high-sensitivity C-reactive protein (CRP) at stool collection (**d**). **a** The interaction between long-term PA and *A. putredinis* abundance in relation to body weight change from age 21 to stool collection. **b** Long-term PA in relation to weight change from age 21 to stool collection among participants with low and high *A. putredinis* abundance separately. Box plot centers show the median with boxes indicating their inter-quartile ranges (IQRs) of each quintile range of long-term PA. **c** Association between long-term PA and body weight change from age 21 to stool collection according to *A. putredinis* abundance. The dots in the plot indicate beta coefficients in the multivariable-adjusted generalized linear mixed-effects regression models, with error bars indicating upper and lower limits of their 95% confidence intervals. Beta coefficients and* p*_interaction_ were calculated from multivariable-adjusted generalized linear mixed-effects regression models while adjusting for age, smoking, Alternative Healthy Eating Index (AHEI), total energy intake, probiotic use, antibiotic use, and Bristol stool scale. **d** Associations between PA measures with other body weight measures, including BMI at stool collection, fat mass percentage at stool collection, short-term body weight change in 6 months (using data of the first pair of stool collections only), plasma HbA1c and CRP at stool collection

We then assessed the top 10 most abundant species for their modifications on the associations that were modified by PCo1 or PCo2 (Supplementary Fig. 8). We found that only *A. putredinis* showed a consistently significant modification effect across the examined associations. Higher abundance of *A. putredinis* significantly strengthened the association of long-term PA with less weight gain from age 21 to stool collection, both when *A. putredinis* was analyzed in continuous (*p*_interaction_ = 3.5 × 10^−4^) (Fig. 3a) and binary forms (Fig. 3b,c). Specifically, in individuals with a higher abundance of *A. putredinis* (relative abundance > 3%, median), each 15 MET-hours/week increment in long-term PA was associated with an average of 2.26 kg (95% CI, 1.53–2.98 kg) less weight gain from age 21 to stool collection, whereas individuals with a low abundance of *A. putredinis* (relative abundance ≤ 3%) only had 1.01 kg (95% CI, 0.41–1.61 kg) less weight gain (*p*_interaction_ = 0.019, Fig. 3c). We used 15 MET-hours/week of PA as the unit since it roughly corresponds to 30 min/day of brisk/very brisk walking, which is the recommended minimum level for health maintenance [23]. *A. putredinis* showed a similar modification on the associations of recent PA with lower BMI (*p*_interaction_ = 3.6 × 10^−4^), lower fat mass percentage (*p*_interaction_ = 6.0 × 10^−5^), less weight gain in 6 months (*p*_interaction_ = 0.008), and lower plasma HbA1c (*p*_interaction_ = 0.038) (Fig. 3d).

When examined by PA intensity, the modification effect of *A. putredinis* was largely consistent across vigorous, moderate, and light PA (Supplementary Fig. 9). Also, we found that the modification effect of *A. putredinis* might be species-specific since other species in Alistipes genus were relatively rare (median relative abundance 0.0–0.6%) compared to *A. putredinis* (median relative abundance 3.0%) and we did not observe such a consistent modification from other species in the genus *Alistipes* (Supplementary Fig. 10).

### *A. putredinis’* metabolic pathways and enzymes driving its modification effect

**Fig. 4:**
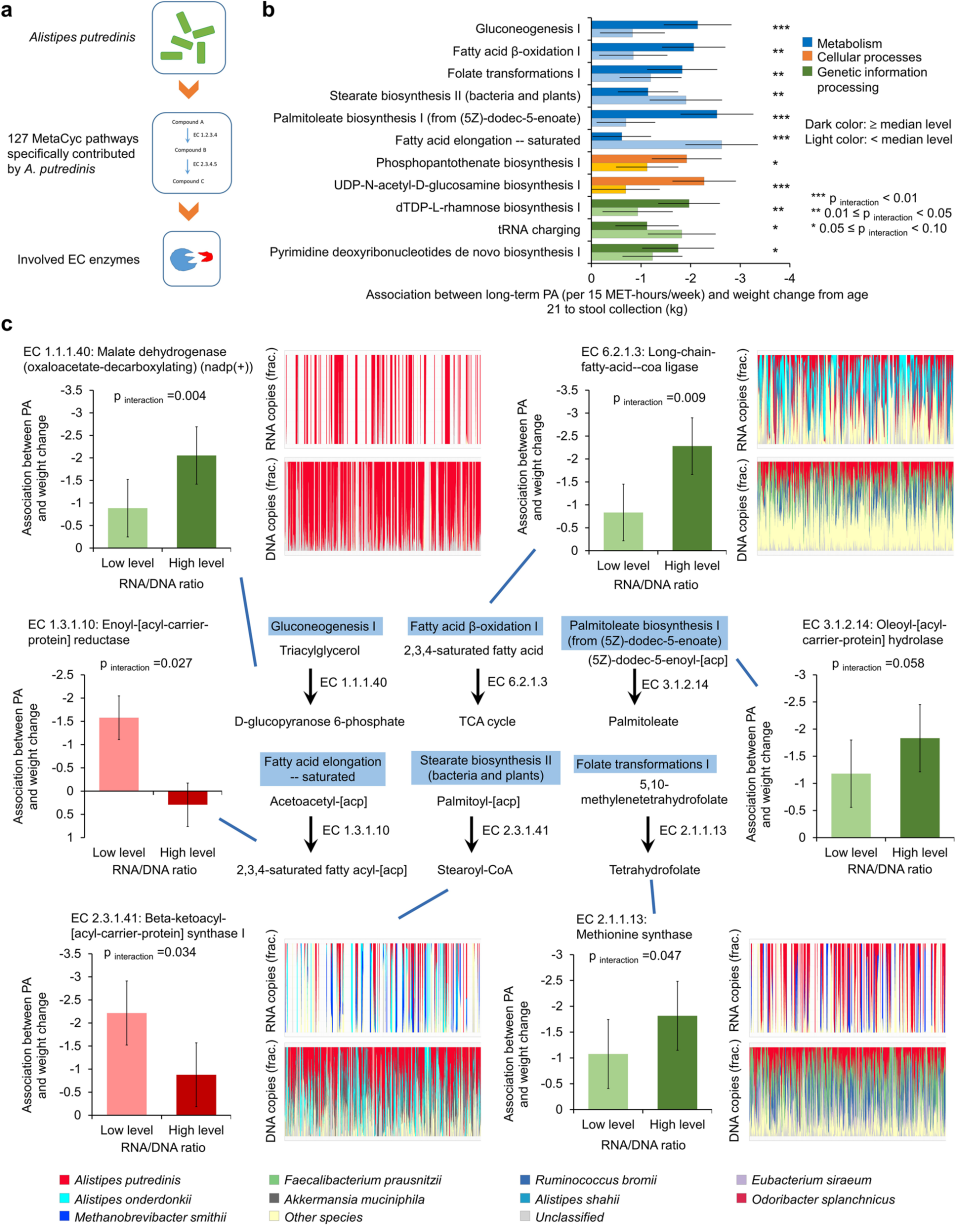
MetaCyc pathways and involved EC enzymes driving the modifying role of *Alistipes putredinis *in body weight response to physical activity (PA). The role of *A. putredinis* in increasing body weight response to PA was mainly driven by the MetaCyc pathways related to gluconeogenesis, fatty acid β-oxidation, palmitoleate biosynthesis, folate transformation, stearate biosynthesis, and fatty acid elongation. **a** Diagram showing that a total of 127 MetaCyc pathways contributed by A. *putredinis* and involved EC enzymes were examined for their modifying roles in the association between PA level and weight change since age 21 years. **b** The 11 MetaCyc pathways modifying the association between PA and weight change since age 21 years. Among the 11 pathways, 6 were of metabolism, 2 were of cellular processes, and 3 were of genetic information processing. The pathways of gluconeogenesis I, fatty acid β-oxidation I, palmitoleate biosynthesis (from (5Z)-dodec-5-enoate), and folate transformations I might positively drive the response in body weight to PA, whereas the pathways of stearate biosynthesis II (bacteria and plant) and fatty acid elongation—saturated might negatively drive the response. The bars indicate beta coefficients in the multivariable-adjusted generalized linear mixed-effects regression models, with error bars indicating upper and lower limits of their 95% confidence intervals. Beta coefficients and *p*_interaction_ were calculated from multivariable-adjusted generalized linear mixed-effects regression models while adjusting for age, smoking, Alternative Healthy Eating Index (AHEI), total energy intake, probiotic use, antibiotic use, and Bristol stool scale. **c** Representative EC enzymes involved in the 6 pathways of metabolism in panel **b** showing modifying role in the association between PA and body weight change since age 21 years, and the contributions of *A. putredinis* to the enzymes. The bar plots in **c** show the microbial species with the greatest contributions to each EC enzyme, with metagenomic or metatranscriptomic samples along the *x*-axes ordered by PA level (from the lowest to the highest). Direction of the modifications of the pathways and detected enzymes were consistent, with the bar chart showing the positive modifications colored in green and negative modifications colored in red. All statistical tests were two-sided

Among the 127 pathways that had contributions from *A. putredinis*, 47 were included after quality control (“Statistical analysis” section). We examined community-level abundances of these 47 pathways and found that 11 of them showed a significant interaction with long-term PA in relation to weight change from age 21 to stool collection (*p*_interaction_ < 0.10), including 6 pathways in metabolism, 2 in cellular processes, and 3 in genetic information processing (Fig. 4b). Of the 6 pathways in metabolism, individuals harboring gut microbiomes with higher abundances of gluconeogenesis I (GLUCONEO-PWY) (*p*_interaction_ = 0.006), fatty acid β-oxidation I (FAO-PWY) (*p*_interaction_ = 0.019), palmitoleate biosynthesis I (PWY-6282) (*p*_interaction_ = 0.001), or folate transformations I (PWY-2201) (*p*_interaction_ = 0.049), or lower abundances of fatty acid elongation – saturated (FASYN-ELONG-PWY) (*p*_interaction_ = 5.2 × 10^−4^) or stearate biosynthesis II (PWY-5989) (*p*_interaction_ = 0.039) showed a stronger association of long-term PA with less weight gain from age 21 to stool collection.

Among the 6 pathways in microbial metabolism, we found that 4 had at least one enzyme with *A. putredinis* as the dominant contributor (top 1st) and the RNA/DNA ratio of these enzymes showed a significant modification on the association of PA with body weight change. These enzymes included EC 1.1.1.40: malate dehydrogenase (oxaloacetate-decarboxylating) (nadp( +)) (*p*_interaction_ = 0.004) in gluconeogenesis I, EC 6.2.1.3: long-chain-fatty-acid–coa ligase (*p*_interaction_ = 0.009) in fatty acid β-oxidation I, EC 2.1.1.13: methionine synthase (*p*_interaction_ = 0.047) in folate transformations I, and EC 2.3.1.41: beta-ketoacyl-[acyl-carrier-protein] synthase I (*p*_interaction_ = 0.034) in stearate biosynthesis II (Fig. 4c). For the pathways of palmitoleate biosynthesis I and fatty acid elongation – saturated, we did not detect any involved enzyme for which *A. putredinis*’ contribution ranked the highest, but we observed some involved enzymes showing a modification effect on the association of PA with weight change since age 21, e.g., EC 3.1.2.14: Oleoyl-[acyl-carrier-protein] hydrolase (*p*_interaction_ = 0.058) and EC 1.3.1.10: Enoyl-[acyl-carrier-protein] reductase (*p*_interaction_ = 0.027) (Fig. 4c).

Taken together, our results support the notion that the modification effect of *A. putredinis* on body weight response to PA may be due to microbial metabolic pathways involved in gluconeogenesis, fatty acid oxidation, folate transformation, and stearate biosynthesis.

### Validation of *A. putredinis’* modifying role in an independent cohort

**Fig. 5:**
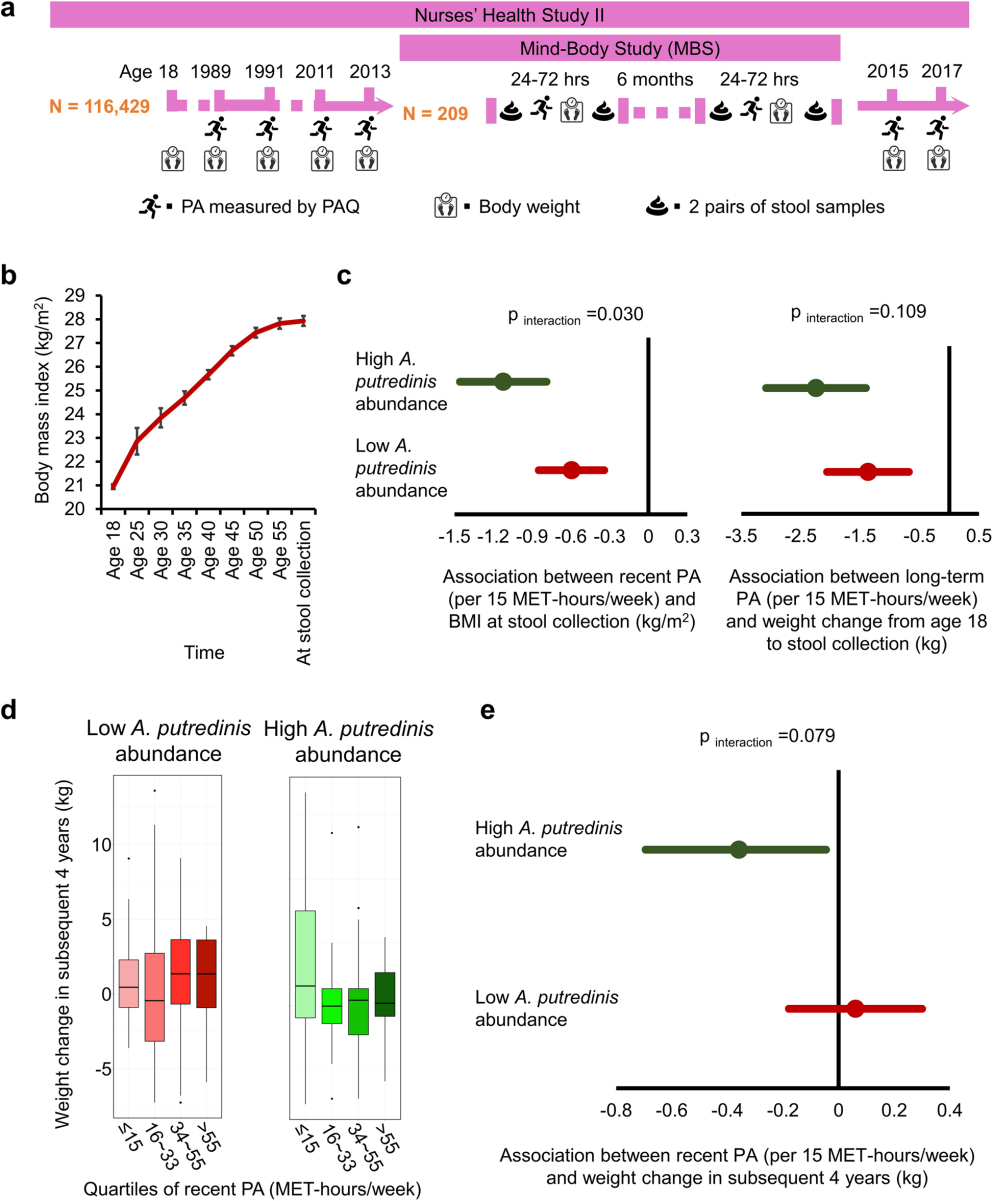
Validation of the modifying role of *Alistipes putredinis* in body weight response to physical activity (PA) in the Mind–Body Study (MBS) cohort. **a** Study design of MBS. To associate the gut microbiome with PA and body weight measures, we profiled stool metagenomes from the MBS. The MBS is a sub-study of the NHSII, an ongoing prospective cohort totaling 116,429 women. The NHSII has repeatedly collected PA and body weight information using validated questionnaires and health-related information since 1989. In the period 2013–2014, the MBS collected stool samples at up to four time points per individual and body weight information at up to two time points 209 participants. We applied MetaPhlAn 2 and HUMAnN 2 to perform taxonomic and functional profiling from stool shotgun metagenomes and metatranscriptomes. **b** Mean value of BMI at different ages and the time of stool collection. The error bars represent standard deviations. **c** Associations between PA level at stool collection and BMI at stool collection (left), and between long-term PA level and body weight change from age 18 to stool collection (right) in those with a higher and lower abundance of *A. putredinis* separately. **d** Body weight change in the subsequent 4 years since stool collection according to PA level at the time of stool collection in those with a higher and lower abundance of *A. putrdinis* separately. **e** Association between PA level at the time of stool collection and body weight change in the subsequent 4 years since stool collection in those with a higher and lower abundance of *A. putrdinis* separately

Mind–Body Study (MBS) is a subcohort nested in the NHS II. Mean (SD) age of MBS participants at stool collection was 60 (3) years. Similar to MLVS, MBS had *A. putredinis* among the top 10 loading microbial species. Using the median abundance of *A. putredinis* as cutoff, we observed that participants harboring a higher abundance of *A. putredinis* had a stronger association of long-term PA with less weight gain since age 18 (− 2.24 kg per 15 MET-hours/week), compared to those with a lower abundance of *A. putredinis* (− 1.37 kg/m^2^ per 15 MET-hours/week) (*p*_interaction_ = 0.109) (Fig. 5c). A similar modification effect of *A. putredinis* was observed on the inverse association between recent PA and BMI (− 1.12 vs. − 0.60 kg/m^2^ per 15 MET-hours/week in those with high vs. low *A. putredinis* abundance) (*p*_interaction_ = 0.030) (Fig. 5c). Notably, when we prospectively examined the body weight change from 2013 to 2017, we found that recent PA level in 2013 was significantly associated with weight loss/less weight gain in the subsequent 4 years only in individuals with a higher *A. putredinis* abundance (− 0.36 kg per 15 MET-hours/week, 95% CI, − 0.69 to − 0.05 kg), but not in those with a lower *A. putredinis* abundance (0.06 kg per 15 MET-hours/week, 95% CI, − 0.18 to 0.30 kg) (*p*_interaction_ = 0.079) (Fig. 5d,e). Therefore, the similar finding in the validation study in younger women supports the presence of a modification effect of *A. putredinis* on body weight response to PA.

## Discussion

Developing effective strategies for body weight management remains a challenge worldwide [1, 2]. Although PA offers a cost-effective way for body weight control [6], there is high inter-individual variability in body weight response to PA [9–17]. In the current study among generally healthy individuals in middle-to-late adulthood, we demonstrated that habitual PA was modestly associated with several microbial species carrying metabolic pathways including glucose metabolism, amino acid metabolism, and SCFA production. More notably, we found robust evidence supporting a modification effect of the specific species *A. putredinis* on body weight response to PA; that is, individuals with a high abundance of *A. putredinis* had a stronger association of PA with less weight gain and reduced levels of obesity-related plasma biomarkers, compared to those with a low abundance of *A. putredinis*. We also observed that the microbial metabolic pathways of gluconeogenesis, fatty acid β-oxidation, palmitoleate biosynthesis, and folate transformations might associate with the modification effect of *A. putredinis* when it is present. Our findings support the consideration of gut microbial features as an important component influencing individuals’ body weight and metabolic responses to PA.

Modification effect by the microbiome indicates phenotypes for which pre-existing microbial differences correspond with changed outcomes (such as weight gain), even if the microbiome itself is not substantially changed by an exposure (such as PA). In the current study, the observed modification effect of gut microbial features on the associations between PA and adiposity measures is striking. Although we cannot address causality because of the observational nature of this study, these findings suggest two main possible hypotheses. First, the carriage of *A. putredinis* may improve the positive effect of PA on controlling body weight gain via changes in gut microbial metabolism. Alternatively, low PA in combination with some additional exogenous factors may increase both the chance of *A. putredinis* acquisition and of weight gain. Further interventional studies are needed to differentiate these alternatives. If confirmed, measures to enrich *A. putredinis* may be considered to enhance the benefit of PA in body weight control. If supported by the appropriate direction of causality, this could potentially be more effective than direct modifications of the microbiome to support weight loss, since we observed no significant association of PA with overall microbial configuration and only weak associations with specific microbial taxa and functions. These latter findings are consistent with prior studies [22, 24–31] and add to the growing evidence that enhanced microbial glucose metabolism and SCFA production may be contributing, but not determining, factors by which PA benefits energy balance.

Several potential modifiers on body weight response to PA have been proposed, e.g., modified appetite, PA performed beyond the prescribed level, and perceived reinforcement value of food [9, 32, 33]. However, inter-individual variability in PA’s effectiveness remains even after adjusting for net exercise energy expenditure and changes in energy intake in an exercise interventional study [11], suggesting the existence of other unrecognized modifiers. Recently, gut hormones were identified as neurotransmitters within the central nervous system to control energy homeostasis [12, 34, 35]. Some gut bacterial metabolites, e.g., SCFA, stimulate the production of gut hormones, e.g., glucagon-like peptide-1 (GLP-1), peptide YY (PYY), and ghrelin, which act in the brain to regulate both food intake and systemic energy expenditure [36]. Given the interplay between the gut microbiome and gut endocrine cells [34, 37], it is plausible that host energy expenditure elicited by PA may be modulated by gut microbial features (independently of dietary energy intake). By further identifying the metabolic pathways and enzymes in *A. putredinis* driving the modification effect and validating the modification effect in an independent cohort, our study provides evidence supporting the gut microbiome, particularly *A. putredinis*, as an important modifier on individual’s response in body weight to PA.

Our finding represents a specific example of the gut microbiome’s broader ability to influence host metabolism through a variety of regulatory and energy balance mechanisms [38]. In the current study, we found that, among the functional pathways expressed by *A. putredinis*, for example, expression of enzymes for folate/methionine transformations showed positive modifications on the association between PA and body weight change. This is only one of several *A. putredinis* activities implicated in the interaction, but it already suggests the variety of pathways by which it could act: changes in extracellular folate pools in the colon signal the host via both energy and immune regulation [39, 40], and microbial regulation in turn is driven by tetrahydrofolate (THF), S-adenosylmethionine (SAM), and other methionine cycle metabolites [41, 42]. Another example of *A. putredinis* pathways positively modifying body weight response to PA is fatty acid β-oxidation. Although gut anaerobes generally do not produce energy by fatty acid β-oxidation [43], the process again modifies both colonic signaling and oxidative stress balance, in ways that can, e.g., help reverse host alcohol-induced energy imbalance and dysbiosis [44]. However, in combination with the several other metabolic pathways in *A. putredinis* showing effect modifications, it is not clear which of these may be causal drivers of the weight change phenotype versus passenger effects of modified *A. putredinis* physiology, and more studies are thus needed.

Once confirmed, our findings can have important implications. First, it supports the potential of gut microbial modulation in bolstering the efficacy of PA in body weight control. Personalized strategies tailored to individuals’ gut microbial features may represent a promising and sustainable approach to mitigate adiposity-related health problems. Second, our findings open a new venue of research in improving the abundance of *A. putredinis* to enhance PA-related metabolic benefit. Although we did not observe a direct association of PA with *A. putredinis*, *A. putredinis* was observed to be increased by PA intervention in prediabetic population [22]. Intakes of cruciferous vegetables were also reported to increase *A. putredinis* abundance in humans [45]. Further studies are needed to elucidate new measures to enrich *A. putredinis* to inform future prebiotic development.

Our study has several strengths, including the assessments of both long-term and short-term PA, the use of accelerometers for a more accurate PA quantification, analysis of deeply phenotyped cohorts with detailed information on multiple body weight and related metabolic measures, and validation of the findings in an independent cohort. Limitations are also noteworthy. First, it was not an interventional study, so we were unable to establish causality between the PA exposures and body weight measures. As an observational study, confounding from other factors influencing the gut microbiome and observed associations cannot be ruled out. However, we adjusted for several such factors, including dietary pattern, which did not essentially change our results. Second, systemic mechanistic explanations for the findings are lacking, particularly as they might be transmitted via host regulatory activities. Although we examined the effect modification by both microbial taxonomy and functions, more studies are needed to link the mechanisms to host outcomes before conclusions can be reached. Third, participants in the discovery cohort were uniformly older, predominantly Caucasian men. However, the consistent results in an independent cohort of younger women indicate the generalizability of our findings.

In conclusion, a higher abundance of *A. putredinis* may strengthen the beneficial association of PA with body weight change; the modifying role of *A. putredinis* may be partly attributable to its functional activities in the gut including folate transformation and fatty acid β-oxidation. Our findings provide evidence for a gut microbiome-based personalized approach for body weight management.

## Materials and methods

### Study population

The Health Professionals Follow-up Study (HPFS) is an ongoing prospective cohort study of 51,529 US male health professionals aged 40 to 75 years at enrollment in 1986 [46]. Participants completed a baseline and biennial follow-up questionnaires to provide comprehensive information on lifestyle factors, medication use, and diagnoses of chronic diseases, with follow-up rates exceeding 90% in each 2-year cycle. The Men’s Lifestyle Validation Study (MLVS) is a sub-study of the HPFS and consisted of 700 men aged 52 to 81 years who were free of coronary heart diseases, stroke, cancer (except squamous or basal cell skin cancer), and major neurological diseases [47]. From 2012 to 2013, a total of 307 men in the MLVS provided up to two pairs of self-collected stool samples from consecutive bowel movements; each pair of samples were collected 24–72 h apart, and the two pairs were collected approximately 6 months apart [47]. The stool collection methods have been detailed and validated previously [47–49]. Two blood samples were drawn, 6 months apart, to coincide with the timing of the fecal sample collection (Fig. 1a).

Nurses’ Health Study (NHS) II is a large, prospective cohort study that enrolled 116,429 registered female nurses aged 25–42 years in 1989. Similar follow-up procedures have been used as in the HPFS cohort. Participants completed a detailed biennial questionnaire regarding their lifestyle and medical history with over 90% of follow-up [50]. Mind–Body Study (MBS), as a subcohort nested in the NHS II, adopted the same protocols for stool sample collection and sequencing as in the MLVS [51]. During 2013–2014, 233 women in MBS were mailed stool sample collection kits and 209 women returned usable stool samples (Fig. 5a). Shotgun sequencing data of the 209 individuals were used for the validation analysis in the current study. Because more comprehensive measurements had been conducted in MLVS than MBS, e.g., accelerometer administration, doubly labeled water test, and blood sample collection, we used MLVS as the discovery cohort and MBS as the validation cohort.

### Assessment of physical activity

In 1986 in the MLVS and 1991 in the MBS and every 2 years thereafter, using a modified Paffenbarger physical activity questionnaire (PAQ), participants reported mean time spent per week engaged in specific activities during the past year, using 13 categories ranging from “None” to “40 + h”. Activities included walking to work or for exercise (including golf), jogging (> 10 min/mile), running (≤ 10 min/mile), bicycling (including stationary machine), lap swimming, tennis, squash or racquetball, calisthenics or rowing, other aerobic exercise (e.g. exercise classes), lower-intensity exercise (e.g., yoga, stretching, or toning), moderate outdoor work (e.g., yardwork or gardening), heavy outdoor work (e.g., digging or chopping), weightlifting (including machines), and standing or walking around work and around home. Participants indicated activity intensity (low, medium, high) for swimming, biking, and tennis. Participants also reported usual walking pace outdoors as easy, average, brisk, or very brisk and stair flights climbed daily. The questionnaire was validated against 4 7-day activity diaries administered across 4 different seasons (correlation, 0.65) and against resting heart rate (correlation, − 0.45) [52]. In the MLVS and MBS studies, two extra PAQs were each administered around 1 month before the 1st and after the 2nd stool sample collection. In the MLVS, from 1986 through 2013, the vast majority of participants provided PA information at all 16 times PAQ (14 times in HPFS and 2 times in MLVS) (92%). To best catch long-term habitual PA level, we calculated the cumulative average PA of the first 15 times and of all 16 times to represent long-term PA level at the time of the 1st and 2nd stool sample collection, respectively.

We used metabolic equivalent task (MET)-hours to represent PA levels. MET is the ratio of work metabolic rate to a standard resting metabolic rate of 1.0 (4.184 kJ) kg^−1^ h^−1^ [53]. A MET value was assigned to each activity adapted from a compendium of physical activities [54]. We derived a measure of MET-hours for each activity by multiplying the MET value by the participant-reported average time using the category midpoint. Total physical activity was defined as the sum of specific MET-hours per week for each activity. Activities were grouped by intensity: vigorous PA (≥ 6 METs: brisk and very brisk pace walking, jogging, running, stairs, squash/racquetball, and high-intensity bicycling, lap swimming, and tennis), moderate PA (3–5.9 METs: average pace walking, moderate/heavy outdoor work, other aerobic exercise, weightlifting and low- to moderate-intensity bicycling, lap swimming, and tennis), and light PA (< 3 METs: easy pace walking, lower-intensity exercise, and other unstructured activities that are distinct from sedentary behavior) [55].

Participants of MLVS were provided an accelerometer (ActiGraph GT3X; Actigraph Corporation, Pensacola, FL), detailed instructions, and a wear time diary. Accelerometers measure body movement during wear and objectively measure PA behavior. At two time points of approximate 1 month before the 1st and after the 2nd stool collection, they were asked to wear the monitor on the hip for 7 consecutive days during waking hours, except bathing or swimming, and maintain a wear time diary. Accelerometer methods have been described elsewhere [56]. We used accelerometer data based on the triaxial vector magnitude. We used the regression equation of [METs = 0.000863* (activity counts from all 3 axes) + 0.668876] to predict METs from triaxial counts [57]. For each minute of non-wear time or when triaxial counts were < 100 per minute, a value of 1 MET was assigned. The computed METs were summed over all minutes to get total energy expenditure in MET-min/day, which was then divided by 60 and 7 to compute MET-hours/week. We used a threshold of 200–2689 counts/minute for light-intensity activity (< 3 METs), 2690–6166 counts/minute for moderate-intensity activity (3–5.99 METs), and ≥ 6167 counts/minute for vigorous-intensity activity (≥ 6 METs) [57, 58]. We derived time spent in each intensity category by summing every minute per day in each intensity level. Accelerometer measures were averaged over valid days for each week of wear. We used the PA level measured by the 2 accelerometers to represent recent PA level each for the time of the 1st and 2nd stool sample collection.

Due to a lack of accelerometer administration in the MBS, we used the PA data collected by PAQ at the time of stool collection to represent recent PA and mean PA across all PAQs in NHS II and MBS to represent long-term PA.

### Assessment of body weight

At the enrollment of HPFS in 1986, participants were requested to report their body weight and height and recall their body weight at age 21. Body weight at age 18 was inquired at enrollment in the NHS II in 1989. Since then, participants reported their body weight biennially through questionnaires. The Pearson correlation coefficient between self-reported body weight and the mean of 2 standardized technician-measured weight was 0.97 [59]. In MLVS, at the time of each of the 2 pairs stool collections, participants additionally reported their current body weight. We calculated body mass index (BMI) by dividing the reported weight in kilograms by the square of height in meters (kg/m^2^) reported in HPFS. We used the difference between the 2 body weight measurements at the two stool collections (2nd minus 1st) to represent short-term body weight change in 6 months, and the difference between the reported body weights at stool collections and age 21 to represent long-term body weight change since age 21. A positive value of the weight change represents weight gain and negative value represents weight loss.

In the MLVS, approximately coincided with the 2 accelerometer administrations, a Doubly Labeled Water (DLW) test was conducted to estimate fat-free mass. Isotope dilution has been long considered as one of the reference methods for the measurements of body composition. It has been well documented that fat-free mass in healthy adults has a hydration of 73% [60]. Knowing the N_O_ from the DLW protocol, total body water was calculated using the equation of total body water = N_O_/1.01 because the N_O_ is assumed to overestimate total body water by 1% [60]. Therefore, fat-free mass was calculated from total body water using the equation of fat-free mass = total body water /0.73. We used the difference between the reported body weight at stool collection and the corresponding fat-free mass as the fat body mass and calculated fat mass percentage as one of our outcome measures.

### Assessment of other covariates

In the HPFS and NHS II, dietary information was collected at the baseline (HPFS: 1986; NHS II: 1991) and updated every 4 years thereafter with validated food frequency questionnaires [61]. In the MLVS and MBS, two extra questionnaires were each administered around the time of stool sample collection to evaluate the diet in the past 1 year. We calculated total energy intake by multiplying the frequency of consumption of each food item by its nutrient content and summing across all foods. Information on smoking status (current smoker or not), use of probiotics in the past 2 months, use of antibiotics in the past 12 months, and the Bristol Stool Chart score (ranging from 1 to 7) was collected through questionnaires completed at the time of sample collection.

### Measurement of plasma biomarkers

Fasting blood samples were collected twice in the MLVS participants, 6 months apart, during the same time as stool collections by nurse practitioners at a clinical laboratory. Participants were cannulated in the forearm (antecubital vein) to collect a blood sample after fasting for 12 h. For each blood sample, information on fasting status, blood collection time and date, smoking status, and body weight was recorded. After collection, blood samples were placed on ice packs, stored in Styrofoam containers, returned to the laboratory via overnight courier, and centrifuged and aliquoted for storage in liquid-nitrogen freezers (− 130 °C or colder). High-sensitivity C-reactive protein (CRP) concentration was determined in plasma by an immunoturbidimetric high-sensitivity assay using reagents and calibrators from Denka Seiken with assay day-to-day variability between 1 and 2%. Hemoglobin A1c (HbA1c) was measured by turbidimetric immunoinhibition using packed red cells (Roche Diagnostics), which is a standard approved by the US National Glycohemoglobin Standardization Program and Food and Drug Administration for clinical use. Our study included 304 participants in the analyses on blood biomarkers. Among 304 participants, 164 and 140 participants provided two and one blood sample, respectively, yielding a total of 468 blood samples. Among the randomly selected 10% quality control (QC) samples, the coefficients of variation were < 7% for CRP and HbA1c. We observed significantly positive correlations of BMI and fat mass percentage with plasma CRP (with BMI 0.49, *p* = 0.004; with fat mass percentage 0.52, *p* = 0.002) and HbA1c levels (with BMI 0.45, *p* = 0.009; with fat mass percentage 0.53, *p* = 0.001) (Supplementary Table 2).

### Taxonomic and functional profiling of metagenomic and metatranscriptomic samples

Taxonomic and functional profiles were generated using the bioBakery meta’omics workflow [62]. Sequencing reads were passed through the KneadData 0.3 quality control pipeline (http://huttenhower.sph.harvard.edu/kneaddata) to remove low-quality read bases and reads of human origin. Taxonomic profiling was performed using MetaPhlAn 2.6.0 (http://huttenhower.sph.harvard.edu/metaphlan2) [63], which classifies the metagenomics reads to taxonomies and yields relative abundances of taxa identified in the sample based on approximately 1 million clade-specific marker genes derived from 17,000 microbial genomes (corresponding to > 13,500 bacterial and archaeal). We excluded microbial species that did not surpass minimum prevalence (10% of samples) and relative abundance (0.01%) threshold.

Shotgun metagenomes and metatranscriptomes were functionally profiled using HUMAnN 2.8.0 (http://huttenhower.sph.harvard.edu/humann) [64]. Briefly, for each sample, taxonomic profiling is used to identify detectable organisms. Reads are recruited to sample-specific pangenomes including all gene families in any detected microorganisms using Bowtie2 [65]. Unmapped reads are aligned against UniRef90 [66] using DIAMOND translated search [67]. Hits are counted per gene family and normalized for length and alignment quality. For calculating abundances from reads that mapped to more than one reference sequence, search hits are weighted by significance (alignment quality, gene length, and gene coverage). UniRef90 abundances from both the nucleotide and protein levels were then mapped to level 4 Enzyme Commission (EC) nomenclature [68] and combined into structured pathways from MetaCyc [69]. More details about functional profiling in the MLVS have been described previously [47, 48].

Metatranscriptomic functional activity was assessed in 341 MLVS samples with both RNA and DNA data using RNA/DNA ratio. Owing to the compositionality of RNA and DNA measurements, the resulting ratio is relative to the mean transcript abundance of the entire microbial community. Thus, a ratio of 1 implies that the pathway is transcribed at the mean transcription abundance of all pathways in the microbial community. Only samples with at least 1 read per kilobase (RPK) were included in the analysis. Infinite values of RNA/DNA ratios were imputed using 99% percentile of a given feature and values of 0 were imputed using half of the 1% percentile before log transformation.

### Statistical analysis

We linked each of the microbiome measurements with the PA, body weight, biomarkers, and other variables collected at the time closest to the time of stool collection. Specifically, the 1st and 2nd metagenomes and metatranscriptomes (i.e., the first pair of stool samples) were linked to recent PA assessed at the first accelerometer, long-term PA assessed at the 1st stool collection, anthropometry assessed at the first questionnaire and DLW test, and biomarkers measured at the first blood sample collection. Similarly, the 3rd and 4th metagenomes and metatranscriptomes were linked to the corresponding second recent and long-term PA, anthropometry, and biomarker measures. We used recent PA level to link with BMI and fat mass percentage at stool collection, short-term weight change in the past 6 months, and plasma CRP and HbA1c at stool collection; and used long-term PA level to link with weight change since age 21.

To investigate variation in species composition, we used principal coordinate analysis (PCoA) for each sample using the Bray–Curtis measure to calculate the dissimilarity matrix. We performed omnibus testing with permutational multivariate analysis of variance (PERMANOVA) to quantify the percentage of variance in the microbial communities explained by PA measures including recent and long-term total PA and PA by intensity, body weight measures including BMI and fat mass percentage at stool collection, short-term weight change in 6 months (restricted in data of the 1st pair stool samples), and long-term weight change since age 21, plasma CRP and HbA1c, and covariates based on the Bray–Curtis dissimilarity metric using the adonis function in the R package vegan 2.5–6. All the PERMANOVA tests were two-sided with the degree of freedom of 1.

For per-feature tests, we first performed quality control filtering for taxonomic and functional features by removing those with a relative abundance of < 0.01% in less than 10% of samples. Similarly, we filtered out all enzymes with a relative abundance of < 0.001% or detected in less than 10% of all samples. After filtering, 139 microbial species were included in the analysis. Using the raw functional profiling abundances calculated for metagenomes and metatranscriptomes, we quantified functional activity of the gut microbial transcripts by calculating the RNA/DNA ratio of microbial enzymes, which provides an index of over/under-transcription (relative to DNA copy number) within each individual microbiome sample [64]. Pathways and enzymes that had < 1 read per kilobase of either RNA or DNA were treated as not detected in this calculation. In addition to the filters of minimum abundance and prevalence, functional features with high correlations with others were removed by taking the most abundant feature in each of such clusters as its representative. We employed the R package MaAsLin 2 1.0.0 to perform per-feature tests (https://huttenhower.sph.harvard.edu/maaslin2) [70]. We log-transformed relative abundances of microbial features before including them in the MaAsLin models. Multivariable models were adjusted for age, smoking, total energy intake, probiotic use, antibiotic use, and Bristol stool scale. In the per-feature tests, unless otherwise noted, all high-dimensional tests were corrected for multiple comparison by controlling the false discovery rate using the Benjamini–Hochberg method with a target rate of 0.25 for *q* values. The circular phylogenetic tree was produced by GraPhlAn as a part of the bioBakery workflow (https://huttenhower.sph.harvard.edu/graphlan).

We used generalized linear mixed-effects regressions (with generalized least squares estimator) for all the association analyses. All models included identifiers of participants as random effects to account for within-subject correlation due to repeated sampling, and included the PA measures and covariables of age, smoking, Alternative Healthy Eating Index (AHEI), total energy intake, probiotic use, antibiotic use, and Bristol stool scale as fixed effects. We firstly carried out hypothesis-generation by testing the potential interactions of PA measures with the 1st and 2nd principal coordinate loading scores (PCo1 and PCo2) of the overall gut community structure in relation to the body weight measures and biomarkers. We included in the models the main effects of the PA measures and PCo1 or PCo2, as well as their product term, in addition to the covariables as fixed effects and participant identifier as random effect. Of the 139 microbial species passing the quality control criteria, the 10 most abundant species together accounted for 46% of the overall community abundance. Thus, on top of a significant interaction between PA and PCo1 or PCo2, we further tested the interactions between PA and relative abundances of the top 10 loading individual microbial species using the same methods. We used the median abundance level as cutoff for each species to dichotomize the samples and examined their interactions with the PA level in relation to body weight measures. We tested the significance level of the beta coefficient of the product term using a two-sided likelihood ratio test by comparing models with and without the interaction term to calculate the *p* value for interaction. A significant *p* value of the product term indicates an interaction between the PA measures and the overall microbial composition or an individual microbial species, referred to as a modification effect. We used the same methods in examining the modification effect of the gut microbiome on the association between PA and body weight in the validation cohort.

After observing the modification effect of *A. putredinis*, we further examined the metabolic pathways contributed by *A. putredinis* and the involved enzymes to understand the mechanism through which *A. putredinis* modulates body weight response to PA. Among all the pathways contributed by *A. putredinis*, we firstly performed a quality control by excluding those correlated with others with a correlation of > 0.9 and taking the most abundant feature in each of such clusters as its representative. For the pathways that showed significant modification effect, we examined all the involved enzymes. For each enzyme, we ranked the contributions from various species in metagenomes and, if the contribution from *A. putredinis* ranked the highest, we then examined the interaction of RNA/DNA ratio with long-term PA in relation to weight change from age 21 to stool collection. Metatranscriptomes were functionally profiled using the same methods as metagenomes in 341 MLVS samples with both RNA and DNA data. Median RNA/DNA ratio was used as the cutoff to define low and high transcriptional activity of *A. putredinis*. All analyses were two-sided and performed using R version 3.6.1.

## Supplementary Information

**Additional file 1: Supplementary Table 1.** Characteristics of participants in the Men’s Lifestyle Validation Study according to quartiles of recent physical activity level. **Supplementary Table 2.** Spearman correlations between the variables of physical activity, body weight, body weight change, and plasma biomarkers. **Supplementary Fig. 1.** Spearman correlation between long-term total physical activity level and calorie-adjusted dietary intakes of major nutrients at *p* < 0.05. **Supplementary Fig. 2.** Principal coordinate analysis of all samples using species-level Bray–Curtis dissimilarity according to recent physical activity level measured by accelerometer. **Supplementary Fig. 3.** Gut microbial diversity using species taxonomic data according to recent total physical activity measured by accelerometer. **Supplementary Fig. 4.** Significant associations of physical activity, body weight measures, and plasma biomarkers of hemoglobin A1c (HbA1c) and high-sensitivity C-reactive protein (CRP) with microbial species (*q* ≤ 0.25). **Supplementary Fig. 5.** Significant associations of physical activity, body weight measures, and plasma biomarkers of hemoglobin A1c (HbA1c) and high-sensitivity C-reactive protein (CRP) with metagenomic pathways (MetaCyc) (*q* ≤ 0.25). **Supplementary Fig. 6.** Significant associations of physical activity, body weight measures, and plasma biomarkers of hemoglobin A1c (HbA1c) and high-sensitivity C-reactive protein (CRP) with metagenomic enzymes (Enzyme Commission, EC) (*q* ≤ 0.25). **Supplementary Fig. 7.** Interaction between physical activity measures and the first two principal coordinates axis (PCo1 or PCo2) in relation to body mass index at stool collection, fat mass percentage at stool collection, short-term (6 months) weight change, long-term weight change from age 21 to stool collection, plasma hemoglobin A1c (HbA1c) and high-sensitivity C-reactive protein (CRP). **Supplementary Fig. 8.** Interaction between physical activity and abundances of the top 10 most abundant species in relation to body mass index at stool collection, fat mass percentage at stool collection, short-term (6 months) weight change, long-term weight change from age 21 to stool collection, plasma hemoglobin A1c (HbA1c) and high-sensitivity C-reactive protein (CRP). **Supplementary Fig. 9.** Associations between intensity-specific physical activity and body weight measures according to *Alistipes putredinis* abundance. **Supplementary Fig. 10.** Relative abundance, prevalence, and interactions of all the species in the genus of *Alistipes* with physical activity in relation to body mass index at stool collection, fat mass percentage at stool collection, short-term (6 months) weight change, long-term weight change from age 21 to stool collection, plasma hemoglobin A1c (HbA1c) and high-sensitivity C-reactive protein (CRP).

## Data Availability

Because of the sensitive nature of the data collected for this study, all the metadata are available through a request for external collaboration and upon approvals of a letter of intent and a research proposal that may be sent to Brigham and Women’s/Harvard Cohorts at https://sites.google.com/channing.harvard.edu/cohortdocs/getting-started/collaborations-consortia?authuser=2]. Source code that generates the figures and tables is available at https://github.com/biobakery/Physical-activity-gut-microbiome-body-weight.
