# Supplementary material for: The gut microbiome modifies the associations of short- and long-term physical activity with body weight changes
Source: Microbiome. 2023 May 30;11:121. doi: 10.1186/s40168-023-01542-w (PMC10228038; doi:10.1186/s40168-023-01542-w)
Supplement: Supplementary file 2 — Additional file 1: Supplementary Table 1. Characteristics of participants in the Men’s Lifestyle Validation Study according to quartiles of recent physical activity level. Supplementary Table 2. Spearman correlations between the variables of physical activity, body weight, body weight change, and plasma biomarkers. Supplementary Fig. 1. Spearman correlation between long-term total physical activity level and calorie-adjusted dietary intakes of major nutrients at p < 0.05. Supplementary Fig. 2. Principal coordinate analysis of all samples using species-level Bray–Curtis dissimilarity according to recent physical activity level measured by accelerometer. Supplementary Fig. 3. Gut microbial diversity using species taxonomic data according to recent total physical activity measured by accelerometer. Supplementary Fig. 4. Significant associations of physical activity, body weight measures, and plasma biomarkers of hemoglobin A1c (HbA1c) and high-sensitivity C-reactive protein (CRP) with microbial species (q ≤ 0.25). Supplementary Fig. 5. Significant associations of physical activity, body weight measures, and plasma biomarkers of hemoglobin A1c (HbA1c) and high-sensitivity C-reactive protein (CRP) with metagenomic pathways (MetaCyc) (q ≤ 0.25). Supplementary Fig. 6. Significant associations of physical activity, body weight measures, and plasma biomarkers of hemoglobin A1c (HbA1c) and high-sensitivity C-reactive protein (CRP) with metagenomic enzymes (Enzyme Commission, EC) (q ≤ 0.25). Supplementary Fig. 7. Interaction between physical activity measures and the first two principal coordinates axis (PCo1 or PCo2) in relation to body mass index at stool collection, fat mass percentage at stool collection, short-term (6 months) weight change, long-term weight change from age 21 to stool collection, plasma hemoglobin A1c (HbA1c) and high-sensitivity C-reactive protein (CRP). Supplementary Fig. 8. Interaction between physical activity and abundances of the top 10 most abundan [file 40168_2023_1542_MOESM1_ESM.docx]

The gut microbiome modifies the associations of short- and long-term physical activity with body weight changes

Supplementary Material

**Supplementary Table 1.** Characteristics of participants in the Men’s Lifestyle Validation Study according to quartiles of recent physical activity level

**Supplementary Table 2.** Spearman correlations between the variables of physical activity, body weight, body weight change, and plasma biomarkers

**Supplementary Figure 1.** Spearman correlation between long-term total physical activity level and calorie-adjusted dietary intakes of major nutrients at p <0.05

**Supplementary Figure 2.** Principal coordinate analysis of all samples using species-level Bray-Curtis dissimilarity according to recent physical activity level measured by accelerometer

**Supplementary Figure 3.** Gut microbial diversity using species taxonomic data according to recent total physical activity measured by accelerometer

**Supplementary Figure 4.** Significant associations of physical activity, body weight measures, and plasma biomarkers of hemoglobin A1c (HbA1c) and high-sensitivity C-reactive protein (CRP) with microbial species (*q* ≤ 0.25)

**Supplementary Figure 5.** Significant associations of physical activity, body weight measures, and plasma biomarkers of hemoglobin A1c (HbA1c) and high-sensitivity C-reactive protein (CRP) with metagenomic pathways (MetaCyc) (*q* ≤ 0.25)

**Supplementary Figure 6.** Significant associations of physical activity, body weight measures, and plasma biomarkers of hemoglobin A1c (HbA1c) and high-sensitivity C-reactive protein (CRP) with metagenomic enzymes (Enzyme Commission, EC) (*q* ≤ 0.25)

**Supplementary Figure 7.** Interaction between physical activity measures and the first two principal coordinates axis (PCo1 or PCo2) in relation to body mass index at stool collection, fat mass percentage at stool collection, short-term (6 months) weight change, long-term weight change from age 21 to stool collection, plasma hemoglobin A1c (HbA1c) and high-sensitivity C-reactive protein (CRP)

**Supplementary Figure 8.** Interaction between physical activity and abundances of the top 10 most abundant species in relation to body mass index at stool collection, fat mass percentage at stool collection, short-term (6 months) weight change, long-term weight change from age 21 to stool collection, plasma hemoglobin A1c (HbA1c) and high-sensitivity C-reactive protein (CRP)

**Supplementary Figure 9.** Associations between intensity-specific physical activity and body weight measures according to *Alistipes putredinis* abundance

**Supplementary Figure 10.** Relative abundance, prevalence, and interactions of all the species in the genus of *Alistipes* with physical activity in relation to body mass index at stool collection, fat mass percentage at stool collection, short-term (6 months) weight change, long-term weight change from age 21 to stool collection, plasma hemoglobin A1c (HbA1c) and high-sensitivity C-reactive protein (CRP)

**Supplementary Table 1. Characteristics of participants in the MLVS according to quartiles of recent PA level**

|  | Quartiles of recent PA measured by accelerometer (MET-hours/week) | | | |
| --- | --- | --- | --- | --- |
| Variable | Q1  (0-15.8) | Q2  (15.9-22.4) | Q3  (22.5-29.9) | Q4  (30.0-73.4) |
| N of individuals (%) | 86 (28) | 79 (26) | 75 (24) | 67 (22) |
| N of stool samples (%) | 230 (25) | 231 (25) | 231 (25) | 233 (25) |
| Age at stool collection, years | 72.6 (5) | 70.9 (4.1) | 70.8 (4.1) | 69.8 (3.2) |
| PA by intensity measured by accelerometer, MET-hours/week |  |  |  |  |
| Vigorous | 0.1 (0.9) | 0.8 (2.4) | 1.9 (4.4) | 5.8 (11.1) |
| Moderate | 3.7 (5.4) | 6 (6) | 10.4 (7.7) | 18.4 (12.2) |
| Light | 10.2 (8.1) | 11.1 (6.9) | 13.8 (10.5) | 14.6 (9.4) |
| Long-term average PA from 1986 to stool collection, MET-hours/week |  |  |  |  |
| Total | 33.2 (20.6) | 46.2 (22.1) | 47.4 (19.9) | 61.4 (27.5) |
| Vigorous | 10.2 (14.4) | 16.5 (17.5) | 18.1 (13.8) | 24.2 (23) |
| Moderate | 15.8 (12.6) | 23 (14.6) | 22.4 (15.9) | 30.4 (20) |
| Light | 3.1 (2.8) | 2.9 (2.4) | 3.3 (2.7) | 3.5 (3) |
| BMI at stool collection, kg/m^2^ | 28.2 (5) | 26.3 (3.6) | 25.2 (3.1) | 24.8 (2.9) |
| Fat mass percentage at stool collection, % | 32.2 (7.6) | 28.4 (6.4) | 27 (5.4) | 26 (6.6) |
| Weight change in 6 months between the 1^st^ and 2^nd^ stool collection, kg | 1.1 (0) | 1.4 (1.6) | 3.6 (0) | 0.5 (0) |
| Weight change between age 21 and stool collection, kg | 13.3 (13.2) | 8 (10.6) | 5.9 (9.4) | 5.3 (8.7) |
| Current smoker at stool collection, % | 2.2 | 2.6 | 1.7 | 1.7 |
| Total energy intake, kcal/d | 1941.2 (465.5) | 2129.2 (481.1) | 2089.0 (448.7) | 2257.5 (498.0) |
| Used probiotics in 2 months, % | 5.2 | 7.8 | 6.9 | 7.3 |
| Used antibiotic in 12 months, % | 26.5 | 28.6 | 29.9 | 19.3 |
| Bristol score, % |  |  |  |  |
| 1-2, hard stool | 15.2 | 10.2 | 16.9 | 10.3 |
| 3-5, normal stool | 70.9 | 71.5 | 65.8 | 73.4 |
| 6-7, loose stool | 13.9 | 16.6 | 17.3 | 16.3 |
| CRP, mg/dL | 2.8 (3.8) | 2 (3.2) | 2.1 (3.4) | 1.4 (2.2) |
| HbA1c, % | 5.8 (0.4) | 5.8 (0.4) | 5.7 (0.4) | 5.7 (0.3) |

Abbreviations: MLVS, Men’s Lifestyle Validation Study; PA, physical activity; BMI, body mass index; CRP, high-sensitivity C-reactive protein; HbA1c, hemoglobin A1c. PA levels were measured by accelerometer. Values are means for continuous variables and percentages for categorical variables. The variable of weight change in 6 months between the 1^st^ and 2^nd^ stool collection was based on the data collected at the 1^st^ stool collection.

**Supplementary Table 2. Spearman correlations between the variables of physical activity, body weight, body weight change, and plasma biomarkers**

| Correlation coefficient | Recent total PA | Recent vigorous PA | Recent moderate PA | Recent light PA | Long-term total PA | Long-term vigorous PA | Long-term moderate PA | Long-term light PA | BMI | Fat mass% | 6-month weight change | Weight change since age 21 | CRP | HbA1c |
| --- | --- | --- | --- | --- | --- | --- | --- | --- | --- | --- | --- | --- | --- | --- |
| p value |  |  |  |  |  |  |  |  |  |  |  |  |  |  |
| Recent total PA | 1 | 0.52 | 0.65 | -0.24 | 0.55 | 0.51 | 0.23 | 0.09 | -0.66 | -0.52 | -0.08 | -0.49 | -0.43 | -0.54 |
|  |  | 0.002 | <.001 | 0.18 | <.001 | 0.002 | 0.20 | 0.62 | <.001 | 0.002 | 0.67 | 0.004 | 0.01 | 0.001 |
| Recent vigorous PA |  | 1 | 0.13 | 0.11 | 0.55 | 0.56 | 0.27 | -0.14 | -0.46 | -0.62 | -0.15 | -0.45 | -0.26 | -0.43 |
|  |  |  | 0.48 | 0.53 | <.001 | <.001 | 0.13 | 0.44 | 0.007 | <.001 | 0.39 | 0.01 | 0.15 | 0.01 |
| Recent moderate PA |  |  | 1 | -0.41 | 0.31 | 0.24 | 0.12 | -0.05 | -0.17 | -0.29 | -0.20 | -0.09 | -0.48 | -0.11 |
|  |  |  |  | 0.02 | 0.08 | 0.18 | 0.52 | 0.77 | 0.35 | 0.10 | 0.27 | 0.62 | 0.01 | 0.54 |
| Recent light PA |  |  |  | 1 | -0.09 | 0.004 | -0.44 | 0.68 | 0.08 | 0.10 | -0.24 | 0.02 | 0.22 | -0.07 |
|  |  |  |  |  | 0.61 | 0.98 | 0.01 | <.001 | 0.64 | 0.56 | 0.19 | 0.89 | 0.22 | 0.71 |
| Long-term total PA |  |  |  |  | 1 | 0.62 | 0.52 | 0.15 | -0.46 | -0.51 | -0.43 | -0.29 | -0.002 | -0.48 |
|  |  |  |  |  |  | <.001 | 0.002 | 0.41 | 0.01 | 0.002 | 0.01 | 0.11 | 0.99 | 0.01 |
| Long-term vigorous PA |  |  |  |  |  | 1 | 0.21 | 0.17 | -0.38 | -0.54 | -0.31 | -0.33 | 0.13 | -0.57 |
|  |  |  |  |  |  |  | 0.23 | 0.36 | 0.03 | 0.001 | 0.08 | 0.06 | 0.47 | 0.001 |
| Long-term moderate PA |  |  |  |  |  |  | 1 | -0.36 | -0.34 | -0.14 | -0.13 | -0.13 | -0.19 | -0.06 |
|  |  |  |  |  |  |  |  | 0.04 | 0.05 | 0.43 | 0.48 | 0.49 | 0.30 | 0.76 |
| Long-term light PA |  |  |  |  |  |  |  | 1 | -0.12 | 0.04 | -0.37 | 0.05 | 0.12 | -0.19 |
|  |  |  |  |  |  |  |  |  | 0.49 | 0.84 | 0.04 | 0.77 | 0.51 | 0.28 |
| BMI |  |  |  |  |  |  |  |  | 1 | 0.67 | -0.05 | 0.83 | 0.49 | 0.45 |
|  |  |  |  |  |  |  |  |  |  | <.001 | 0.79 | <.001 | 0.004 | 0.01 |
| Fat mass% |  |  |  |  |  |  |  |  |  | 1 | 0.25 | 0.56 | 0.52 | 0.53 |
|  |  |  |  |  |  |  |  |  |  |  | 0.15 | 0.001 | 0.002 | 0.001 |
| 6-month weight change |  |  |  |  |  |  |  |  |  |  | 1 | -0.32 | 0.07 | -0.09 |
|  |  |  |  |  |  |  |  |  |  |  |  | 0.07 | 0.71 | 0.63 |
| Weight change since age 21 |  |  |  |  |  |  |  |  |  |  |  | 1 | 0.26 | 0.26 |
|  |  |  |  |  |  |  |  |  |  |  |  |  | 0.15 | 0.15 |
| CRP |  |  |  |  |  |  |  |  |  |  |  |  | 1 | 0.22 |
|  |  |  |  |  |  |  |  |  |  |  |  |  |  | 0.22 |
| HbA1c |  |  |  |  |  |  |  |  |  |  |  |  |  | 1 |
|  |  |  |  |  |  |  |  |  |  |  |  |  |  |  |

Abbreviations: PA, physical activity; BMI, body mass index; CRP, high-sensitivity C-reactive protein; HbA1c, hemoglobin A1c. PA level measured by accelerometer at stool collection was used to represent recent PA, and average PA level based on the questionnaires from 1986 to 2012 was used to represent long-term PA.


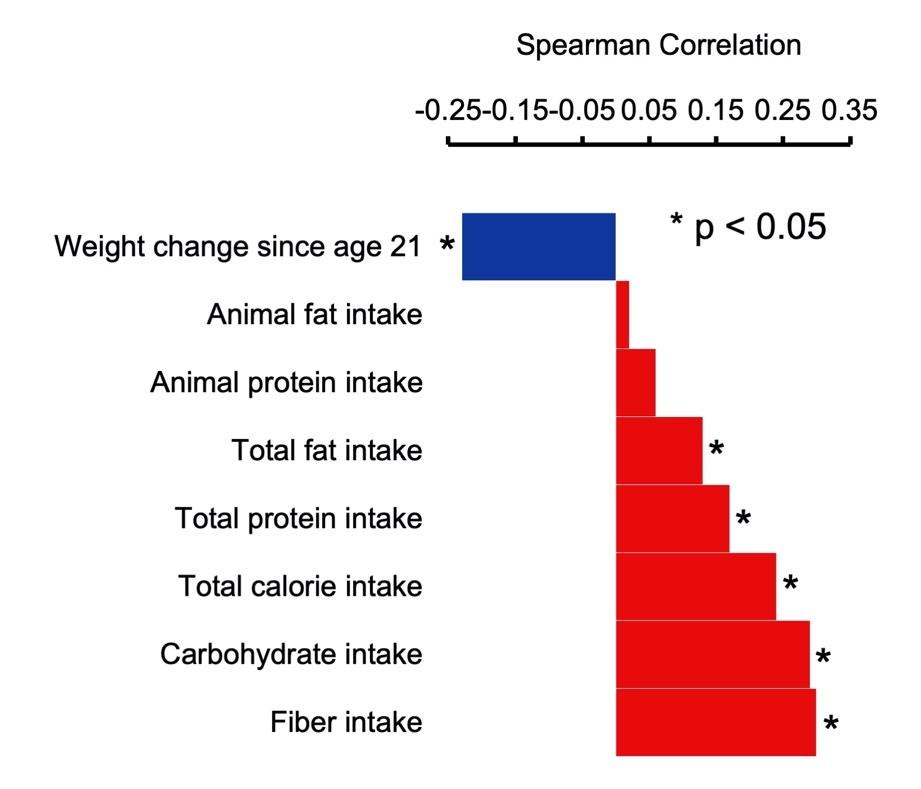


**Supplementary Figure 1. Spearman correlation between long-term total physical activity (PA) level (MET-hours/week) and calorie-adjusted dietary intakes of major nutrients at p <0.05**


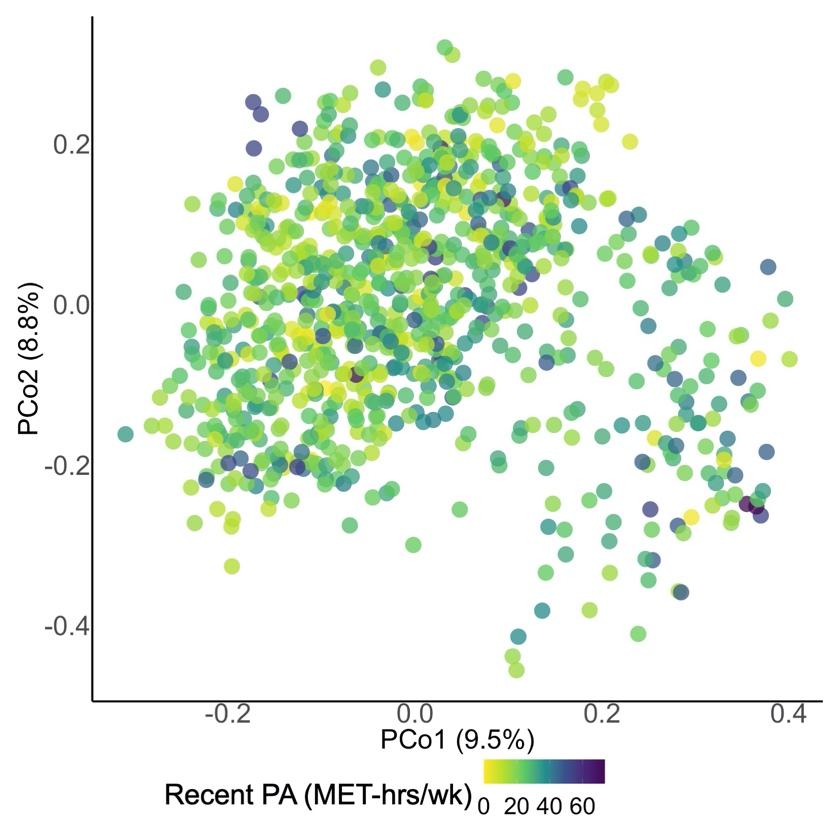


**Supplementary Figure 2. Principal coordinate (PCo) analysis of all samples using species-level Bray-Curtis dissimilarity according to recent physical activity (PA) level measured by accelerometer**


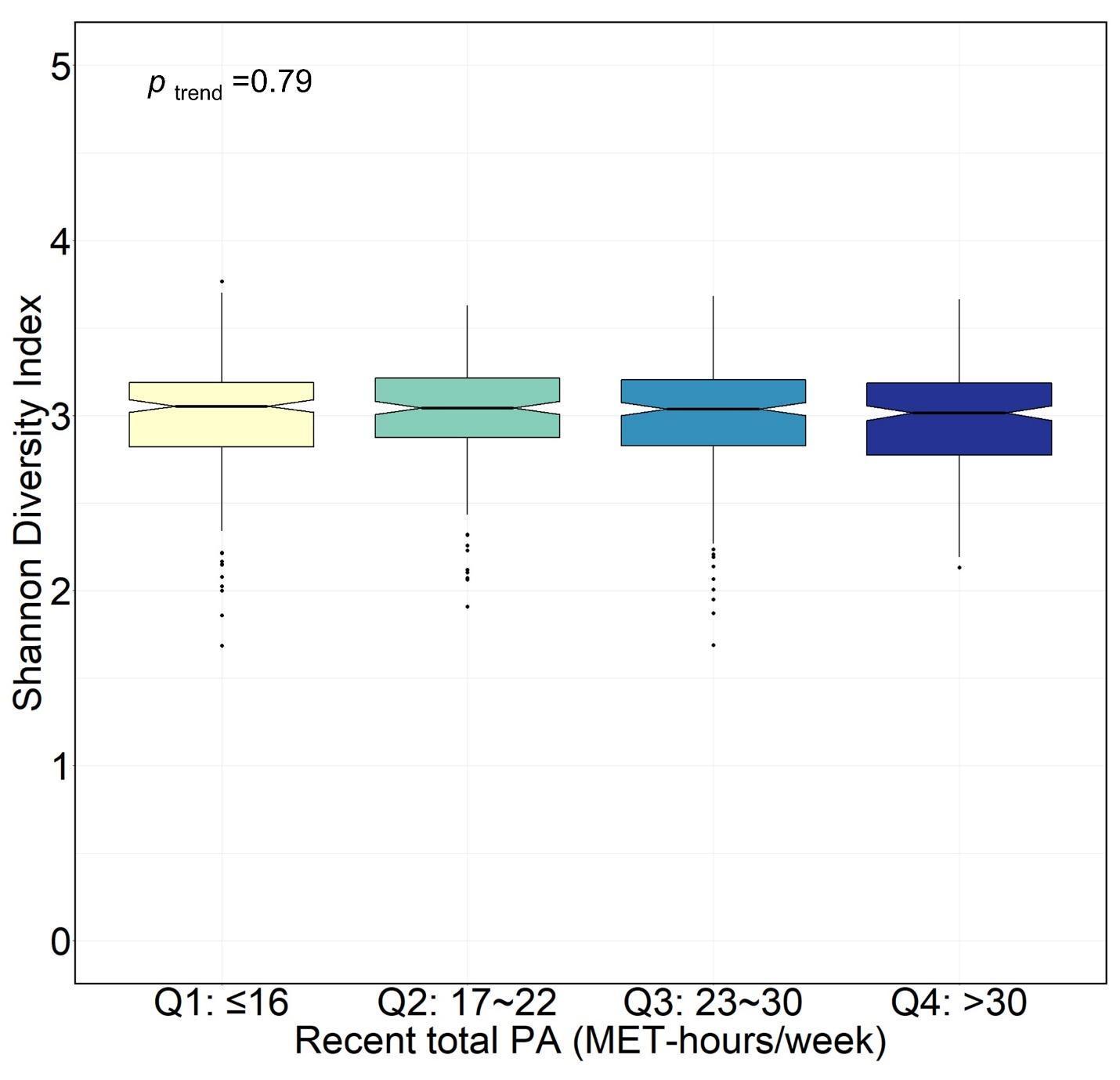


**Supplementary Figure 3. Gut microbial diversity using species taxonomic data according to recent total physical activity measured by accelerometer**


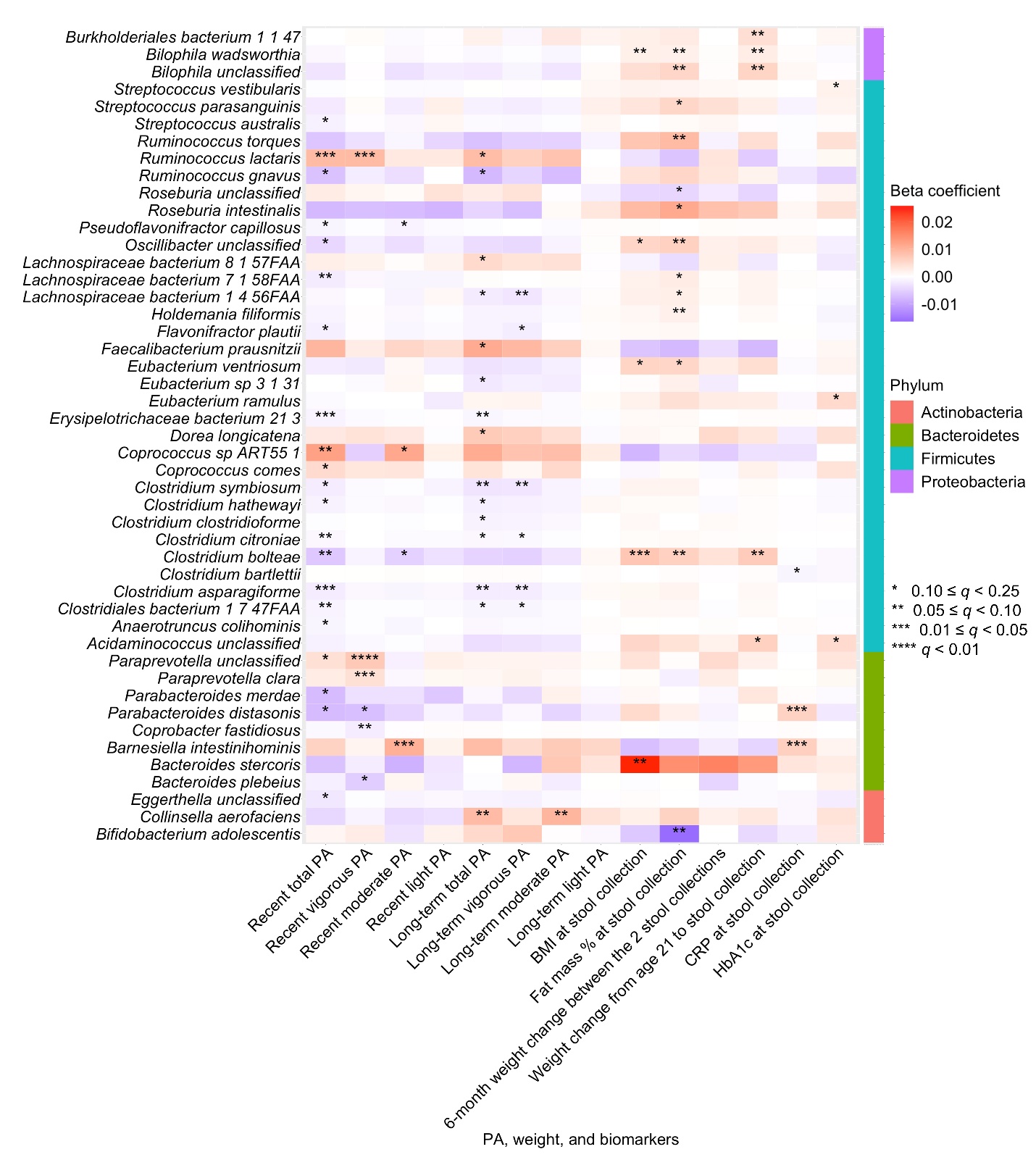


**Supplementary Figure 4. Significant associations of physical activity (PA), body weight measures, and plasma biomarkers of hemoglobin A1c (HbA1c) and high-sensitivity C-reactive protein (CRP) with microbial species (*q* ≤ 0.25)**. The *q* values (false discovery rate adjusted P value) were calculated using the Benjamini Hochberg method with a target rate of 0.25. This plot shows associations of the factors with specific microbial species overlaid onto their taxonomy. The red-to-blue gradient represents the magnitude and direction of the associations between the factors and species’ abundances. All models included each participant’s identifier as random effects and simultaneously adjusted for age, smoking, total energy intake, probiotic use, antibiotic use, and Bristol stool scale.


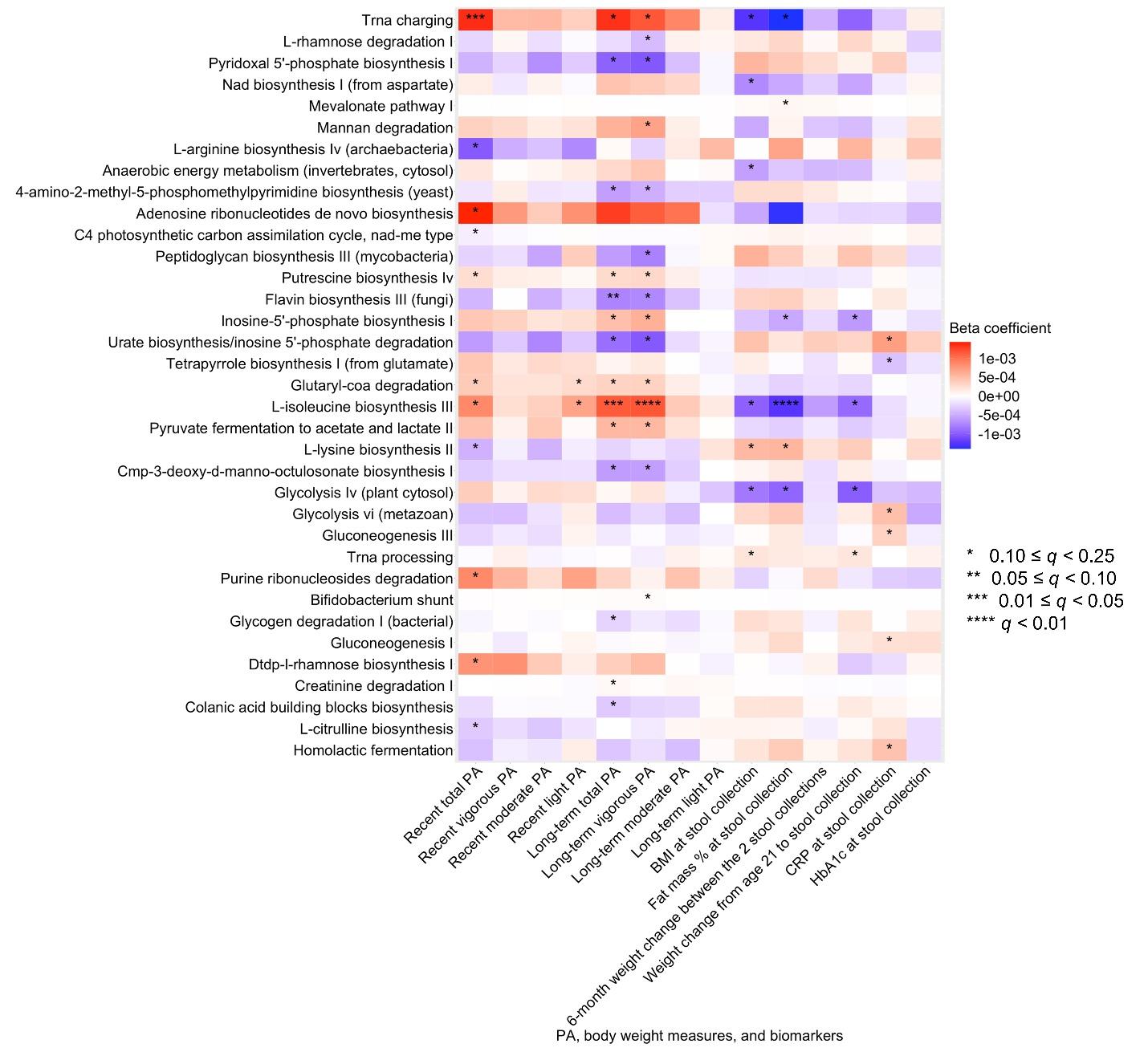


**Supplementary Figure 5. Significant associations of physical activity (PA), body weight measures, and plasma biomarkers of hemoglobin A1c (HbA1c) and high-sensitivity C-reactive protein (CRP) with metagenomic pathways (MetaCyc) (*q* ≤ 0.25)**. The *q* values (false discovery rate adjusted P value) were calculated using the Benjamini Hochberg method with a target rate of 0.25. This plot shows associations of the factors with specific metagenomic pathways. The red-to-blue gradient represents the magnitude and direction of the associations. All models included each participant’s identifier as random effects and simultaneously adjusted for age, smoking, total energy intake, probiotic use, antibiotic use, and Bristol stool scale.


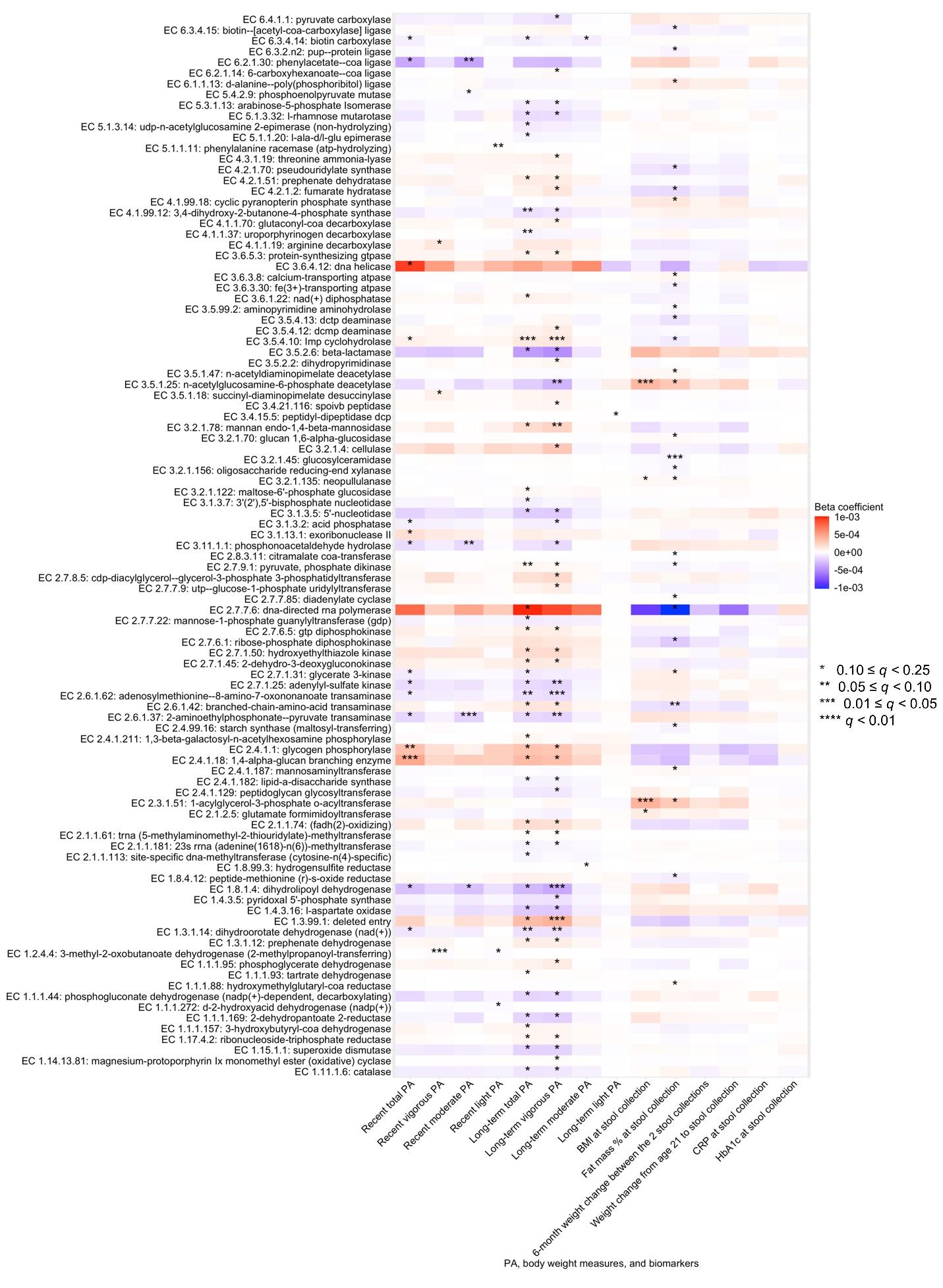


**Supplementary Figure 6. Significant associations of physical activity (PA), body weight measures, and plasma biomarkers of hemoglobin A1c (HbA1c) and high-sensitivity C-reactive protein (CRP) with metagenomic enzymes (Enzyme Commission, EC) (*q* ≤ 0.25)**. The *q* values (false discovery rate adjusted P value) were calculated using the Benjamini Hochberg method with a target rate of 0.25. This plot shows associations of the factors with specific metagenomic enzymes. The red-to-blue gradient represents the magnitude and direction of the associations. All models included each participant’s identifier as random effects and simultaneously adjusted for age, smoking, total energy intake, probiotic use, antibiotic use, and Bristol stool scale.


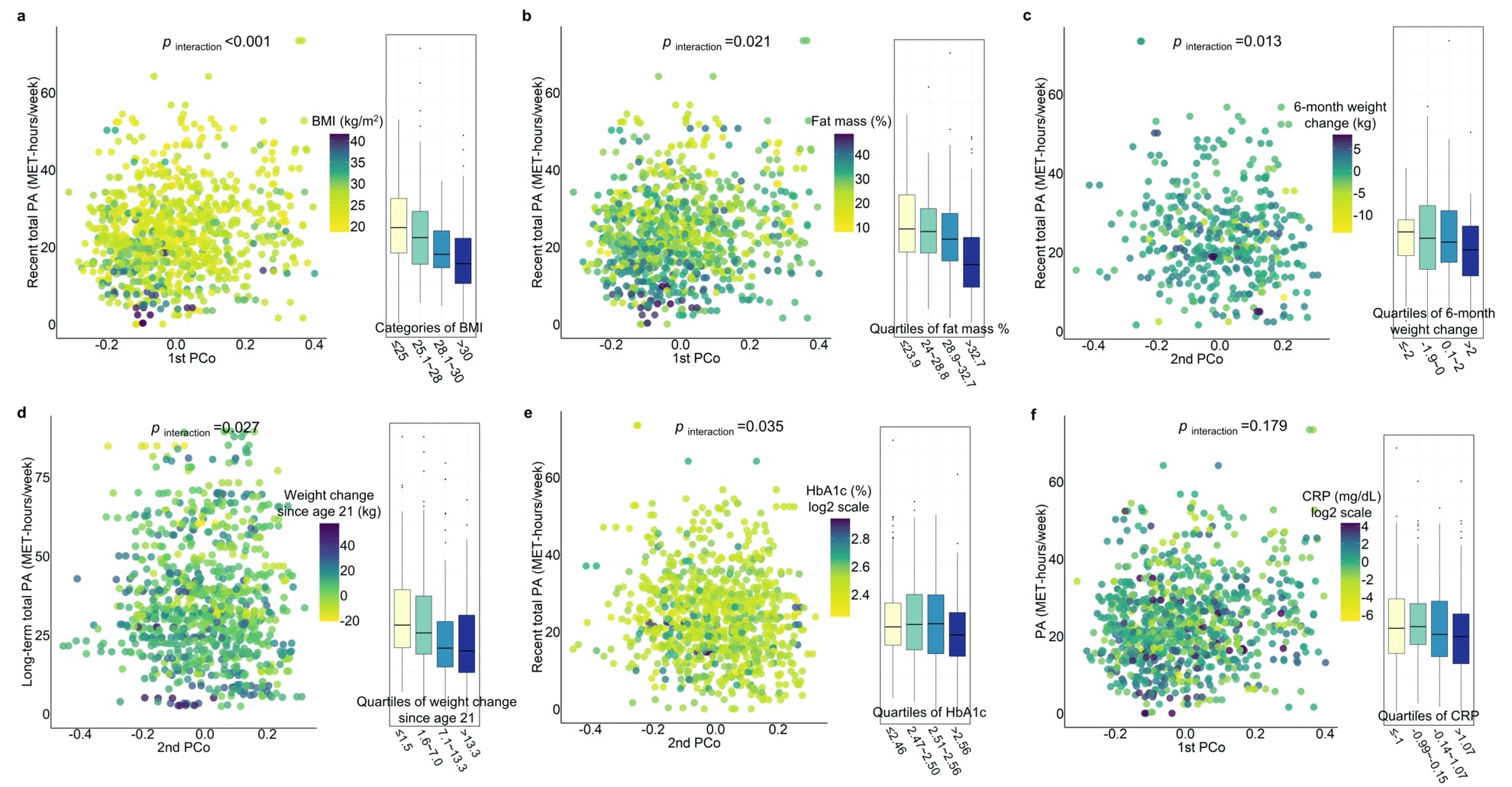


**Supplementary Figure 7. Interaction between physical activity (PA) measures and the first two principal coordinates axis (PCo1 or PCo2) in relation to body mass index (BMI) at stool collection, fat mass percentage at stool collection, short-term (6 months) weight change, long-term weight change from age 21 to stool collection, plasma hemoglobin A1c (HbA1c) and high-sensitivity C-reactive protein (CRP)**. *p* _interaction_ was calculated from multivariable-adjusted generalized linear mixed-effects regression models with each participant’s identifier as random effects while adjusting for age, smoking, Alternative Healthy Eating Index (AHEI), total energy intake, probiotic use, antibiotic use, and Bristol stool scale.


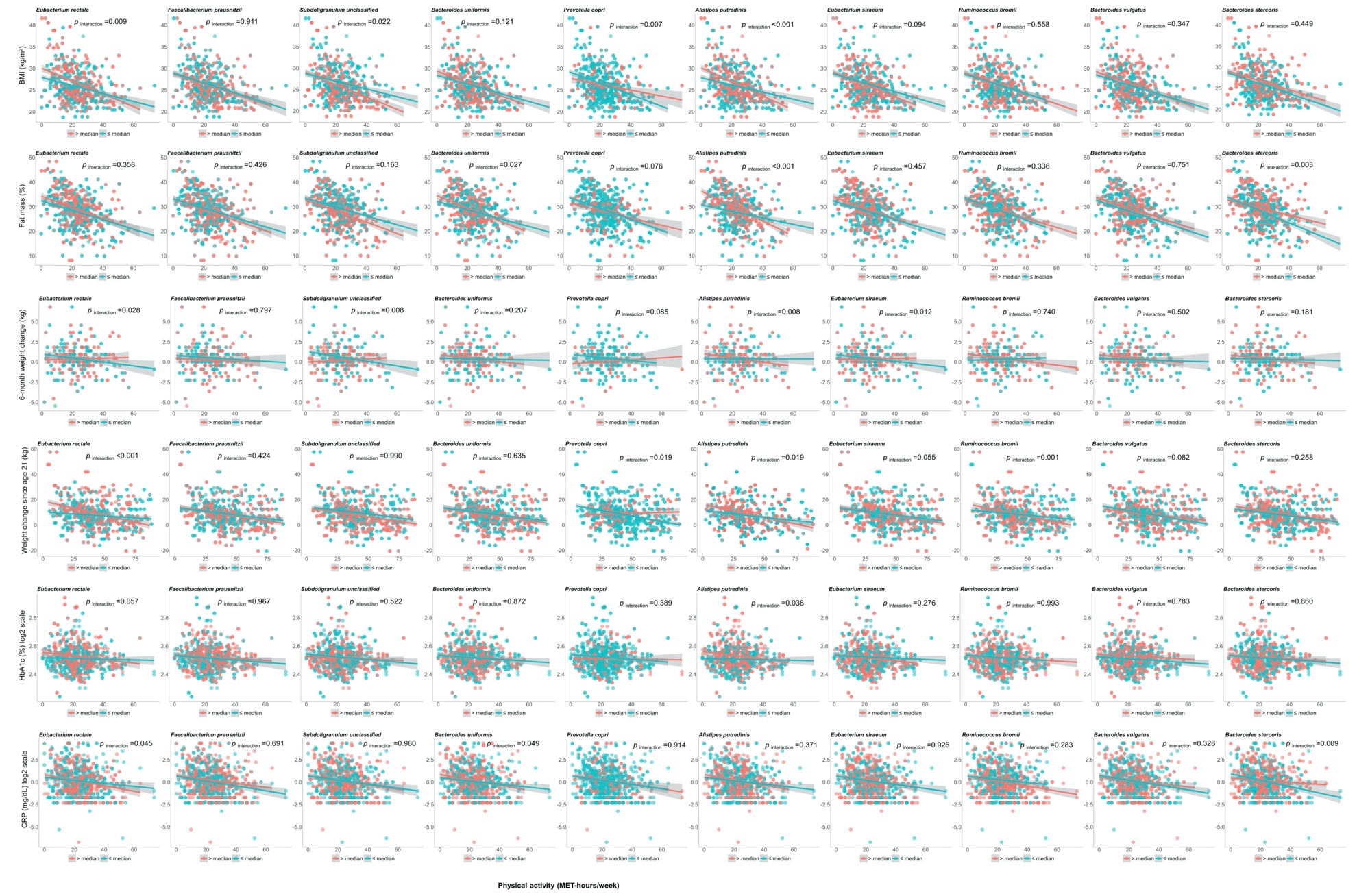
**Supplementary Figure 8. Interaction between physical activity and abundances of the top 10 most abundant species in relation to body mass index (BMI) at stool collection, fat mass percentage at stool collection, short-term (6 months) weight change, long-term weight change from age 21 to stool collection, plasma hemoglobin A1c (HbA1c) and high-sensitivity C-reactive protein (CRP)**. Median abundance of each species was used as cutoff for low and high level. *p* _interaction_ was calculated from multivariable-adjusted generalized linear mixed-effects regression models with each participant’s identifier as random effects while adjusting for age, smoking, Alternative Healthy Eating Index (AHEI), total energy intake, probiotic use, antibiotic use, and Bristol stool scale.


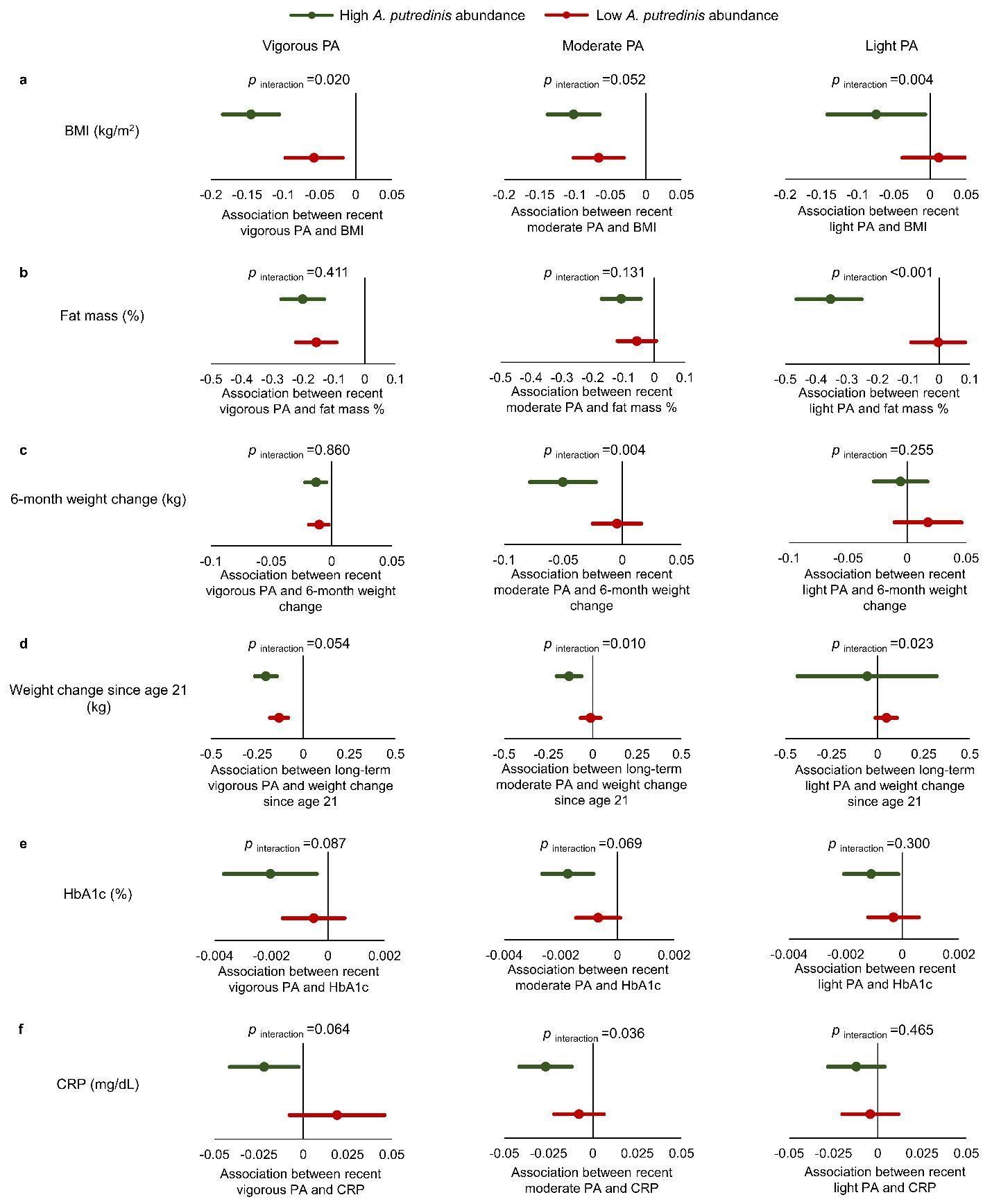


**Supplementary Figure 9. Associations between intensity-specific physical activity (PA) and body weight measures according to *Alistipes putredinis* abundance.** Median abundance of *A. putredinis* was used as cutoff for low and high level. **a**, **b**, **c**, **d**, **e**, and **f** shows the association of PA measures with body mass index (BMI), fat mass percentage (%), 6-month weight change, weight change from age 21 to stool collection, plasma hemoglobin A1c (HbA1c), and high-sensitivity C-reactive protein (CRP), respectively. The dots in the plot indicate beta coefficients in the multivariable-adjusted generalized linear mixed-effects regression models, with error bars indicating upper and lower limits of their 95% confidence intervals. Beta coefficients and *p* _interaction_ were calculated from multivariable-adjusted generalized linear mixed-effects regression models with each participant’s identifier as random effects while adjusting for age, smoking, Alternative Healthy Eating Index (AHEI), total energy intake, probiotic use, antibiotic use, and Bristol stool scale.


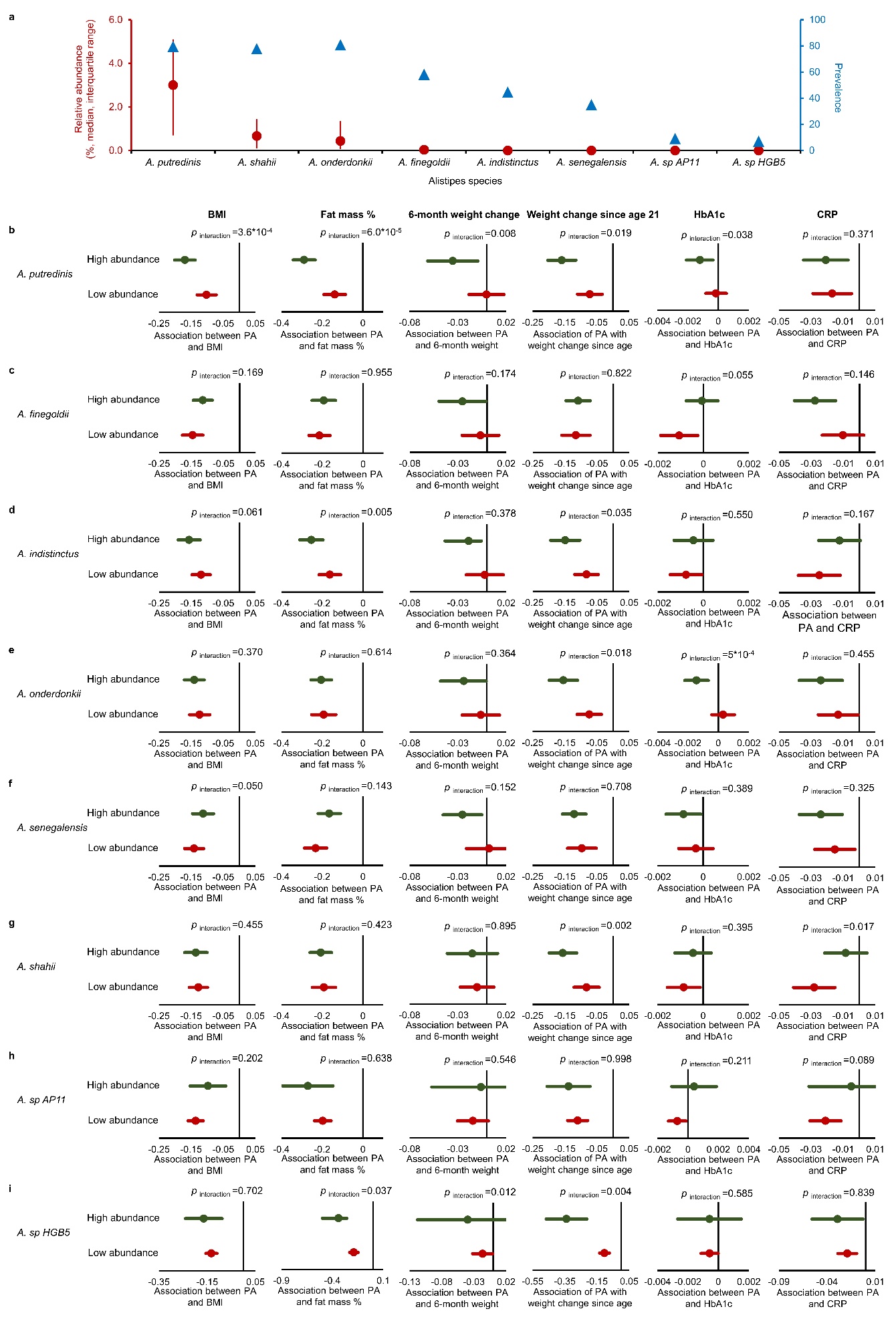


**Supplementary Figure 10. Relative abundance, prevalence, and interactions of all the species in the genus of *Alistipes* with physical activity (PA) in relation to body mass index (BMI) at stool collection, fat mass percentage at stool collection, short-term (6 months) weight change, long-term weight change from age 21 to stool collection, plasma hemoglobin A1c (HbA1c) and high-sensitivity C-reactive protein (CRP).** Median abundance of *A. putredinis* was used as cutoff for low and high level. The dots in the plot indicate beta coefficients in the multivariable-adjusted generalized linear mixed-effects regression models, with error bars indicating upper and lower limits of their 95% confidence intervals. Beta coefficients and *p* _interaction_ were calculated from multivariable-adjusted generalized linear mixed-effects regression models with each participant’s identifier as random effects while adjusting for age, smoking, Alternative Healthy Eating Index (AHEI), total energy intake, probiotic use, antibiotic use, and Bristol stool scale.
